# A Gene-Based Algorithm for Identifying Factors That May Affect a Speaker’s Voice

**DOI:** 10.3390/e25060897

**Published:** 2023-06-02

**Authors:** Rita Singh

**Affiliations:** Center for Voice Intelligence and Security, Carnegie Mellon University, Pittsburgh, PA 15213, USA; rsingh@cs.cmu.edu

**Keywords:** FOXP2, genetic microdeletion syndromes, voice biomarkers, voice chains, voice profiling

## Abstract

Over the past decades, many machine-learning- and artificial-intelligence-based technologies have been created to deduce biometric or bio-relevant parameters of speakers from their voice. These voice profiling technologies have targeted a wide range of parameters, from diseases to environmental factors, based largely on the fact that they are known to influence voice. Recently, some have also explored the prediction of parameters whose influence on voice is not easily observable through data-opportunistic biomarker discovery techniques. However, given the enormous range of factors that can possibly influence voice, more informed methods for selecting those that may be potentially deducible from voice are needed. To this end, this paper proposes a simple path-finding algorithm that attempts to find links between vocal characteristics and perturbing factors using cytogenetic and genomic data. The links represent reasonable selection criteria for use by computational by profiling technologies only, and are not intended to establish any unknown biological facts. The proposed algorithm is validated using a simple example from medical literature—that of the clinically observed effects of specific chromosomal microdeletion syndromes on the vocal characteristics of affected people. In this example, the algorithm attempts to link the genes involved in these syndromes to a single example gene (FOXP2) that is known to play a broad role in voice production. We show that in cases where strong links are exposed, vocal characteristics of the patients are indeed reported to be correspondingly affected. Validation experiments and subsequent analyses confirm that the methodology could be potentially useful in predicting the existence of vocal signatures in naïve cases where their existence has not been otherwise observed.

## 1. Introduction

Aside from diseases that affect the biological structures and processes involved in voice production, myriad other factors are known to influence voice. Some simple examples include age, exhaustion, smoking, which often makes the voice sound hoarse, or alcohol, which makes the voice sound slurred. The ensuing changes in the voice signal can be thought to be the “biomarkers” that give us information about the corresponding causative factors and allow us to infer their nature through voice analysis. Such relationships form the basis for artificial-intelligence (AI)-based voice profiling techniques that attempt to deduce a speaker’s bio-relevant and environmentally related parameters from voice. However, virtually all research on voice profiling, diagnostics, and biometrics is currently predicated on clinically observed or statistically inferred relationships between changes in voice and the corresponding factors that are thought to cause them. The relationships that are chanced upon in this manner provide the basis for building predictive AI (machine-learning- or rule-based) mechanisms that can deduce the underlying factors that potentially influence voice through voice analysis.

For example, it is known that smoking affects voice. To establish this, a *human-observation-based approach* would be: (a) an audiological one based on hearing the voices of smokers to determine if they deviate from those of non-smokers in an acoustic sense, and/or (b) a visual one where an analyst studies the spectrogram (or some other visual representation) of the speech signal to find patterns that distinguish one class of recordings from another. The spectrogram in this case is a “feature representation”. A *statistical approach*, on the other hand, would gather examples of speech recordings from people who smoke and those who do not, extract feature representations from the recordings, and find significant differences in the statistics of these features obtained from the two sets of recordings. Alternatively, a classifier model may be trained to discriminate between voice samples from smokers and non-smokers. If high test accuracies are achieved in this task, the existence of a biomarker for smoking in voice is indicated. This is a purely data-driven approach for establishing the existence of biomarkers in voice.

The problem with these approaches is that neither is scalable. The number of factors that can influence the human persona is virtually infinite. Human observations are limited to the effects that are perceptually discernible in voice, and data-based discovery is confined and limited by the availability of representative data. This paper provides a more formal methodology for establishing the existence of biomarkers and for identifying which factors are likely to affect voice and which are not. The methodology is based on genomic considerations, as explained below.

Before we proceed, however, it must be noted that this is *not* an algorithm for computational biology research. Its use to aid biological discovery or establish biological facts in its current form has not been tested. It is *only* meant to establish the tentative links that are needed to justify computational profiling efforts.

### 1.1. A genomic-Based Approach to Detect the Existence of Biomarkers

The working hypothesis for this paper, and one that has also recently been proposed in the context of voice profiling [1], is that if a given factor exerts an influence on the speaker, and if pathways of biological effects can be traced from that influence to the speaker’s voice production system, then voice *must* be affected (and must carry biomarkers for the factor). The methodology proposed herein is a literal test of this hypothesis in that it traces biological pathways from cause to effect to establish the existence of biomarkers.

For this, we begin with the genetic underpinnings of human vocal capabilities. In this context, it is important to differentiate between voice production and speech production. The former refers to the production of acoustic energy in any form within the vocal tract, and the latter refers to the modulation of the acoustic signals thus produced to form words and sentences in a language used for interpersonal communication. We will use the term “vocal production” to refer to both.

Vocal production in humans is a complex and multifaceted process and involves interactions between multiple genes and environmental factors. The genetic basis of vocal production is not fully understood. Nevertheless, a number of genes have been found to be involved in the process. Some that are now known to influence vocal production include:

1. **FOXP2**: This gene codes for a protein called “Forkhead Box P2”, which is involved in the development and function of the brain, including the areas responsible for language and speech.
2. **TAFT**: This gene codes for a protein called TAFT1, which is involved in the development and function of the larynx, a structure in the throat that is involved in vocal production.
3. **OTOF**: This gene codes for a protein called Otoferlin, which is involved in the development and function of the auditory system, including the inner ear. Feedback from this system greatly influences vocal production.
4. **MYO15A**: This gene codes for a protein called Myosin XVa, which is involved in the development and function of the auditory system, including the hair cells in the inner ear.
5. **SEMA3A**: This gene codes for a protein called Semaphorin 3A, which is involved in the development and function of the auditory system, including the auditory nerve.

Recent efforts to identify and delineate the genes responsible for functional speech in humans have especially highlighted the importance of FOXP2, one of the protein-coding genes mentioned above. It is involved in a variety of biological pathways and cascades that are thought to regulate language development. It is autosomal dominant, and mutations in it cause speech and language disorders (OMIM: SPCH1). In this paper, we choose FOXP2 as an example gene to work with. This choice was only made for illustrative purposes. The methodology presented in its context is itself a generic one and can be applied to any of the genes listed above (and possibly others that exist) whose functions are relevant to the analysis at hand.

For illustrative purposes, we proceeded with the broad and simplifying assumption that any influence on speech and language is ultimately the phenotypic expression of FOXP2. The objective was thus to:

- Formalize the methodology to find a link between an influencing factor and this gene;
- Validate the methodology;
- Demonstrate its predictive potential.

To accomplish these goals, for simplicity, we chose the example of a category of medical conditions for which the underlying genetic causes are known; the effects of the conditions are observed and reported in medical literature, and the effects can involve problems with vocal production.

The medical conditions we choose are chromosomal microdeletion syndromes, which result from the deletion of specific genes in specific cytogenetic locations on human chromosomes. We propose an algorithm to find a link between the genes in the regions of microdeletions, to FOXP2. The connections in these links are derived using a path-search algorithm applied to a graph composed from known biological pathways that involve these (and other) genes. The “strength” of these connections is then defined in terms of the characteristics of the linkage discovered. In the validation stage, our goal is to show that there is a direct correlation between the strength of linkages found and the extent of vocal symptoms experienced by the affected individuals.

Before we proceed with the algorithm, in the paragraphs below, we first provide a working categorization of speech disorders, as reported in various medical literature. This is necessary for the clarity of the results presented later in this paper.

### 1.2. Anomalies in Speech Production

From a bio-mechanical perspective, human speech is the result of two complex processes that happen simultaneously: one that produces sound—the pressure wave that we sense as the voice signal—and another that modulates this signal (through articulator movements) to produce speech, thus altering the voice signal’s frequency characteristics and shaping it into sounds with unique identities that are uttered sequentially to form words and sentences in a language. The overall process of voice and speech production is driven and controlled by neuromuscular and cognitive factors to different degrees. It is also moderated to different degrees by feedback obtained through auditory pathways. Generally, diseases that affect these functions, naturally also influence speech and alter the characteristics of the voice signal to proportional degrees. In most cases, when reporting such changes, references to “speech” implicitly include voice, as we see below.

Changes in speech are categorically described in terms of six major aspects of speech production: respiration, phonation, articulation, resonance, evolution, and prosody. In addition, terminology that relates to *voice quality* is often used to describe speech. Voice quality is, however, a subjective term and comprises many constituents, or sub-qualities (e.g., nasal, breathy, rough, twangy, etc.), that refer to the perceptual flavor of speech (or how a speaker’s voice sounds to the listener). Physical anomalies that affect the shape and tissue structure of the vocal tract cause changes in all of these aspects. Speech delays and language difficulties result from cognitive and learning disabilities. These and other intellectual disabilities affect articulation, evolution, and prosody. Their effect on voice also manifests as changes in voice quality. Craniofacial anomalies affect the physical dimensions of the vocal tract structures, often restricting the movement of the articulators as a result and causing speaking impairments. Motor problems affect articulation, phonation, and respiration. These cause speech aberrations and also affect voice quality. Hearing problems disturb the feedback mechanisms involved in controlling speech production and often lead to difficulties in prosody and articulation of speech.

In this paper, we do not focus explicitly on voice acoustic or quality characteristics, focusing instead on problems with speech (that subsume voice characteristics to some extent) as described under the OMIM (referring to the catalog Online Mendelian Inheritance in Man: https://www.omim.org/ (accessed on 21 September 2022)) category “Speech and Language disorders (SPCH1)”. Even this category, however, is too broad and encompasses a wide range of speech problems, such as delays in acquisition of speech abilities, retardation in speech development with age, speech anomalies resulting from language delay, expression, and articulation.

For the purpose of this paper, it is necessary to make finer distinctions between these categories. The problem in doing so is that the language used in the literature to describe voice- and speech-related problems in the context of genetic syndromes is not standardized. For example, the terms “speech disorder”, “speech disturbance”, “speech anomalies”, “speech aberrations”, and “speech impairment” may each refer to a range of symptoms that may be overlapping to various degrees. For the purpose of this paper, it is therefore useful to map the broad range of speech problems into the following categories that are sufficiently discriminatory in terms of the different aspects of speech production mentioned above while still being limited in number:

1. **Absence of speech:** Phrases (in clinical/scientific literature) referring to (a) no development of speech capabilities, (b) no expressive speech, which is mostly limited to vocalizations, or (c) almost absent speech with a severely limited vocabulary (0–4 words).
2. **Apraxia:** Phrases referring to difficulty using language correctly while speaking, leading to speaking and communication difficulties.
3. **Delayed speech:** Phrases referring to developmental delay, the retarded development of the ability to speak, or the retarded acquisition of language skills and communication skills (ability to use a vocabulary correctly to communicate in a cogent manner).
4. **Dysarthric speech or dysarthria**: Phrases referring to speaking problems resultant from damaged, paralyzed, or weakened muscles of the articulators caused by motor problems. Dysarthria results in slurred words, poor phonation, etc. The speaker uses vocabulary as in normal speech but finds it difficult to move the articulators (tongue, lips, jaw, etc.) correctly to form the proper sounds to utter the words.
5. **Idiosyncratic speech:** Phrases referring to poor conformance to cogent language or incoherent language with articulation abnormalities.
6. **Impaired speech:** Phrases referring to poor articulation and phonation, as well as difficulties that result in sparse and disfluent speech.

## 2. Methodology

As mentioned earlier, we consider the example of syndromes resulting from chromosomal microdeletions and focus on their symptoms relating to speech abilities.

Chromosomal microdeletions are structural anomalies of chromosomes in which small sections of a chromosome are deleted or missing. The loss of the specific set of genes from the deleted section often results in phenotypic changes. An *implicated gene* is a gene in the deleted region of a chromosome that is known to cause much of the observed effects of the syndrome in affected individuals. These are identified through microarray and other studies. For most microdeletion syndromes, some such genes have been identified and reported in the medical literature. We use this information from the medical literature as-is in the sections below.

Although the human genome is very large in comparison to a typical chromosomal microdeletion region, microdeletions often cause serious problems. In fact, only a small set of deletions are compatible with life or fetal survival. This set continues to expand with the addition of newly discovered deletions in surviving individuals who have the means to reach genetic testing facilities. However, it is still a very small set and can be exhaustively studied. Most known deletions are well documented in the literature, both from the genetic and medical perspectives. Information about the genes associated with them is readily available through well-curated publicly accessible repositories. Thus, they are good example cases for this paper.

The methodology proposed herein analyzes ensembles of biological pathway *chains*, each of which connects a specific gene in the cytogenetic region of chromosomal microdeletion to the FOXP2 gene. A “biological pathway” here is defined as in standard terminology, referring to a physiological process at the cellular level that is enabled by the action of multiple genes that perform specific functions within the process.

We define a *pathway chain* as the sequential linkage of pathways where links between pathways are shared genes (implicitly meaning that the molecules resultant from genes are shared—we will use the term “gene” with this implicit meaning in the context of pathways for generality going forward). For example, consider a pathway that signals for a cell to stop dividing when an injury to the nuclear DNA strand is being repaired. It may involve the coordinated chemical action of molecules that are formed by the transcription of multiple genes that perform different functions. It would *also* be connected to a repair pathway by necessity. Thus, the two pathways can be considered to be links in a single pathway *chain* (they must share some genes in a functional sense) that perform the function of relaying messages from one pathway to another. Such genes may also perform other functions that are essential to both pathways.

The hypothesis we make here is that for a gene, if a chain from its pathway(s) (i.e., from the biological pathways it contributes to directly) extends to pathways that influence voice production, then the phenotype resultant from the absence or aberrant functioning of the gene can be expected to include anomalies in speech production and voice characteristics.

### 2.1. Voice Chains

Our definition of a *voice chain* extends our definition of a pathway chain in that the head of the chain must now necessarily be a pathway that includes a gene that influences voice or speech production, while the termination of the chain is not necessarily a biological pathway but could include any given set of genes with a common characterization (such as a common cytogenetic location or function).

**Figure 1:**
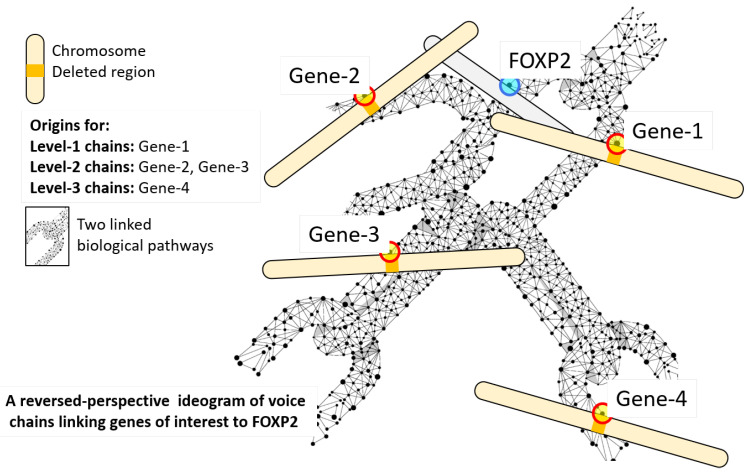
Voice chains of different levels. In this reversed perspective ideogram, a link depicts a pathway, the black dots on it are genes, and the chromosomes that they belong to are shown as rods where relevant. The lines connecting genes are only meant for visual clarity. Chains are formed with respect to genes on a chromosome (a microdeletion region in this case, shown shaded in yellow). In this ideogram, Gene-1 and FOXP2 lie on the same pathway, contributing to a level-1 voice chain. In a level-2 chain, FOXP2 and the microdeletion region are on different pathways, but the pathways share a set of genes. Gene 2 and Gene 3 have level-2 voice chains.

**Figure 2:**
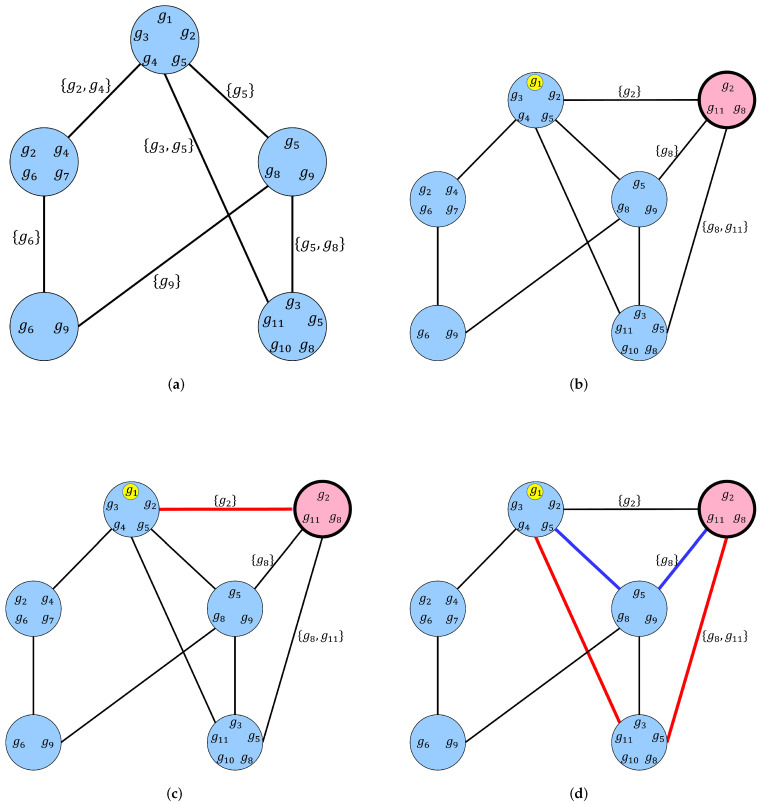
Voice chains of different types. (**a**): Shows the different components of a graph comprising biological pathways. Each node in this graph represents a biological pathway. The genes $g_{i}$ that contribute to each pathway are listed within the node. Nodes are linked to each other through edges that represent the set of shared genes. If two nodes have no gene in common, no edge exists between them. Genes that link pathways (nodes) are explicitly shown on the edges. (**b**): Explains what a chainlink gene means in the context of a microdeletion region and the target voice gene (FOXP2 in the example used in this paper). To compose voice chains, the set of genes of interest (e.g., from a microdeletion region) is added to the a graph as a node (shaded pink). The “chainlink” genes that link the set to the graph are also shown. The topmost (left) node represents a biological pathway that contains the FOXP2 gene ($g_{1}$, highlighted in yellow). (**c**) A Level-1 voice chain is the edge shown in red. The “chainlink” gene $g_{2}$ that links the set (in pink) to the voice chain is also shown. (**d**) Exemplifies Level-2 chains. The colored edges represent Level-2 chains. The “chainlink” genes are also shown.

In this paper, the voice-related gene chosen is FOXP2, but in other analyses, voice chains could involve other genes (e.g., as in [2]) without loss of generality. The terminal link in the chain is taken to be a genetic microdeletion syndrome. A voice chain thus establishes a relationship between an influencing factor—a genetic microdeletion syndrome in this case—and a corresponding effect on voice/speech production. We refer to a voice chain that includes a sequence of α pathways from the microdeletion region to the FOXP2 gene as a *level-α* voice chain (Figure 1). The specific genes on the microdeletion that link it to the voice chain are referred to as “chainlink” genes. We represent the set of chainlink genes that connect a microdeletion to level-α voice chains as the chainlink set $V_{N\alpha }$. Since we aim to analyze the genetic basis of the effect of microdeletions on voice, these chainlink sets are the focus of our analysis. Note that the subscript *N* in $V_{N\alpha }$ denotes the *manner in which* overlaps or linkages between biological pathways are defined. This subscript is fixed for the purpose of this paper, for which the exact manner in which pathway overlaps are defined is described in Figure 1 and Figure 2 below. We however leave the *N* in place to facilitate future differentiations and variations of the proposed algorithm based on how the pathway intersections (or unions) may be defined.

In order to trace the genetic links between a microdeletion syndrome and voice, we first attempt to identify voice chains of different lengths that link to the genes in the microdeletion region. For this, we must find voice chains that link the FOXP2 gene to the syndrome, and identify the specific genes from the syndrome through which they are linked. We do so using the graph-search algorithm described below.

From our perspective, a biological pathway B is represented by the set of genes it involves: B:={g:gisageneinthespecifiedpathway}. Two pathways, $B_{1}$ and $B_{2}$, are *linked* if there are genes that are common to both pathways, i.e., $B_{1}\cap B_{2}\neq \emptyset$. Thus, the set of all pathways can be represented as a graph where the nodes are biological pathways, and two nodes are linked only if the corresponding pathways have common genes, as illustrated in Figure 2a.

A pathway *chain* is any non-repeating sequence of pathways $B_{1}B_{2}B_{3}\cdots B_{N}$ such that $B_{i}\cap B_{i+1}\neq \emptyset$ and $B_{i}\neq B_{j}$ for i≠j, i.e., where every pair of adjacent pathways has common genes, and there are no closed loops in the chain. In terms of the graph (see Figure 2a), a pathway chain is any path between any two nodes in the graph. A *voice chain*V is any chain $V=B_{V}B_{2}B_{3}\cdots B_{N}S$ where the *head* node $B_{V}$ (and the head node alone) is a pathway that includes the FOXP2 gene, i.e., $FOXP2\in B_{V}$, and the terminal node S is a set of genes with common characterization, as mentioned earlier. The *length* of the chain |V| is the number of nodes α in the chain, not counting the terminal node S. For the purpose of this paper, we will assume S to be the set of genes in a microdeletion region associated with a syndrome. Thus, S={g:gisageneinthemicrodeletionregion}. All voice chains of length α form the set of level-α voice chains, and the chainlink genes that connect S to the level-α voice chains form the chainlink set $V_{N\alpha }$.

To find voice chains of the form $B_{V},B_{1},\cdots S$ arising from the microdeletion region S (which we will refer to as the “syndrome” for brevity), we introduce the microdeletion region in the pathway graph (Figure 2b). Voice chains are now the paths from $B_{V}$ to S (Figure 2c,d). A breadth-first algorithm, described in Algorithm 1, is used to extract the chainlink sets $V_{N_{\alpha }}$ for voice chains of multiple levels. The outcome of the algorithm is the set of chainlink genes $V_{N\alpha }\left[S\right]$ that connect each syndrome S to voice chains of level α, for 1≤α≤2. We restrict ourselves to chains of lengths of up to 2 since, at greater lengths, the chained influences cannot be disambiguated, as indicated by prior studies in the (highly related) context of protein–protein interactomes, e.g., [3]. Another reason for restricting ourselves to level 2 chains is that for the specific example chosen in this paper, there are not enough data that allow us to build deeper chains meaningfully (without resorting to self-loops, which may lead to incorrect conclusions).    
**Algorithm 1:** Pseudocode for a breadth-first algorithm for computing the set of chainlink genes that form level 1 and level 2 voice chains for FOXP2.
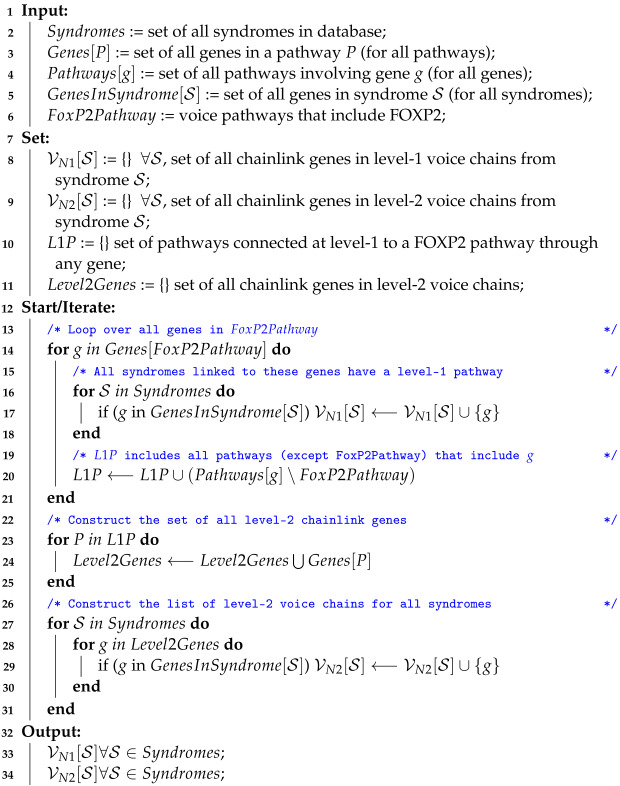


### 2.2. Ensemble Analysis

In the methodology we propose, for any microdeletion region S, we derive the set of chainlink genes within it for which α-level chains exist. The size and composition of this set can then be used in conjunction with the level of the voice chain to indicate the effect on voice (in a later analysis). In general, we can work with any level-α voice chains in such an analysis; however, we restrict ourselves to α=1 and α=2.

## 3. Analysis

$V_{N\alpha },$ where α=1,2, were computed for a total of 82 microdeletion syndromes of chromosomes 1–20/22/X,Y. Genomic information, including gene names, was obtained from the HUGO Gene Nomenclature Committee’s (HGNC) human genome database, comprising 42,764 gene symbols and names and 3245 gene families and sets as of the time of conducting this analysis. Information about the phenotypes and the specific genes implicated in a syndrome was obtained from a survey of the current literature on medical genetics and genomics and from the Online Mendelian Inheritance in Man (OMIM) repository for authoritative information about human genes and genetic phenotypes.

The FOXP2 gene chosen for this analysis has been strongly implicated in speech and language disorders [4,5], including monogenic speech disorders. The cytogenetic location (chromosome locus) of this gene is 7q31.1. Mutations in this gene are known to cause speech and language disorder Type 1, also called “Autosomal dominant speech and language disorder with orofacial dyspraxia”. The phenotype description and known molecular basis for this disorder can be found under OMIM entry SPCH1:602081. The FOXP2 gene encodes for the protein “Forkhead Box Protein P2” [6]. This protein is a transcription factor; it controls the activity of other genes. It binds to the DNA of the genes that it controls through a region known as a Forkhead Domain. It thus plays a critical role in several protein-coding and other biological pathways and has been well studied [7]. A more detailed summary of this gene can be obtained from the Human Protein Atlas [8].

The ensemble of pathways used for this analysis was obtained from the Carcinogenic Potency Database (CPDB), described on its website as “a single standardized resource of the results of 45 years of chronic, long-term carcinogenesis bioassays”. Its current database of human biological pathways contains 4319 pathways and their gene compositions. This database has been used extensively in the medical literature and was chosen in this case for illustrative purposes since there is (importantly) no inherent bias towards the speech phenotype in it. In this database, there is only one pathway that contains the gene FOXP2. This is the Adenoid Cystic Carcinoma (ACC) pathway, which contains 63 genes, listed below for reference: 

**Gene membership of the ACC pathway:**

AKT1 ARID1A ARID4B ARID5B ATM ATRX BCOR BCORL1 BRCA1 BRD1 CEBPA CMTR2 CNTN6 CREBBP CTBP1 DTX4 EP300 ERBB2 ERBIN FBXW7 FGF16 FGFR4 FOXO3 FOXP2 H1-4 H2AC16 HRAS IL17RD INSRR JMJD1C KANSL1 KAT6A KDM6A KDM6B KMT2C MAGI1 MAGI2 MAML3 MAP2K2 MAX MGA MORF4L1 MYB MYBL1 MYC MYCBP MYCN NCOR1 NFIB NOTCH1 NSD1 PIK3CA PRKDC PTEN RAF1 SETD2 SMARCA2 SMARCE1 SMC1A SRCAP TLK1 TP53 UHRF1 

Table A1, given in Appendix A, documents the voice chains found for a set of 75 documented microdeletion syndromes. This range excludes chromosome 21, for which sufficient documentation was not found in the literature. Only voice chains up to level 2 are shown in this table and used in the analysis presented in this paper. This is sufficient to demonstrate the viability of the methodology for the discovery of voice chains proposed in this paper. The entries in the rows and columns of this table are explained in detail in Appendix A.

Table 1 summarizes some of the information in Table A1 to help understand the analysis given in the next section. The information given in Table 1 includes, for each syndrome listed in it, the corresponding implicated genes that are *also* discovered to be chainlink genes by the algorithm proposed in this paper; the overall counts of level-1 and level-2 chainlink genes for each syndrome, along with the number of additional pathways they collectively connect to (in parentheses); and the corresponding phenotypic effects on speech that have been reported in the scientific literature.

## 4. Inferences

A wealth of conclusions can be drawn from Table 1 (and from its more detailed version, Table A1 in Appendix A). However, we focus only on those that help validate the usefulness of the proposed algorithm.

### 4.1. Voice Chains as Predictors of Speech Characteristics

Of the 76 syndromes in Table A1, voice chains were found to exist for all. By our hypothesis, this would imply that in all cases, there is a potential for voice to be affected. The syndromes 15q11–q13 and Xq28 have two versions each, divided in the medical literature based on symptoms, rather than gene composition of the microdeletion region. We can therefore combine them for analysis, leaving us with 74 syndromes to be analyzed. For the syndrome 16q22, no information about the speech issues was found in the medical literature. Only the remaining 73 syndromes are considered in the analysis below.

The incidence of speech pathologies (including *all* forms of pathologies) among the general population is reported to be about 5% [65], and between 2.3% and 24.6% among children [66]. Of the 73 syndromes, 17 syndromes had both level-1 and level-2 voice chains, while 56 had only level-2 chains. The occurrence of speech aberrations was reported for all 17 syndromes with level-1 chains and for all but 6 of the 56 syndromes with only level-2 voice chains. Thus, voice chains correlate highly with the existence of speech anomalies.

### 4.2. Voice Chains as Information-Carrying Entities

Let us study how voice chains correlate with the presence or absence of *specific voice problems*. Such correlations would show that voice chains carry information about how the voice may be affected. This information is expected to be coarse-grained since we only take the presence or absence of any gene into consideration and consider no other cytogenetic information related to it.

From Table A1, we observe the following.

**Level-1 chains:**

The number of level-1 chainlink genes is limited to 1 or 2 in all cases and is not amenable to statistical analysis. However, we make the following observations:

1. Level-1 voice chains co-occur with speech problems 100% of the time.
2. For all instances where level-1 voice chains are present, severe symptoms occur 100% of the time (impaired, delayed or absent speech).
3. For all instances of syndromes with no effect on speech (i.e., where normal is not just one of a range of other speech symptoms), level-1 chains are absent 100% of the the time.

**Level-2 Chains:**

Level-2 voice chains are present in all cases and co-occur with speech disorders in all but 6 cases; thus, in only 6 cases has speech been reported to be normal. Therefore, level-2 voice chains co-occur with speech problems 91.8% of the time.

We note that a syndrome may have level-2 voice chains through many chainlink genes, which could number in the tens or even hundreds. Each of the chainlink genes could, in turn, also be associated with multiple other pathways, in addition to the one connecting it to FOXP2. We refer to the total number of pathways that include the chainlink genes of a syndrome as its “chainlink connectivity”.

Table 2 presents some statistics of syndromes, chainlink genes, and chainlink connectivity associated with speech disorders of different severity.The problems considered are: absent speech (the most severe symptom), impaired speech (a symptom that is less severe than absent), delayed speech (a cognitive symptom that is also less severe than absent and comparable in severity to impaired speech—a physical symptom), dysarthric speech (a symptom related to physical issues), and apraxic speech (due to CNS disorders; this is less severe compared to absent speech and often subsumes idiosyncratic speech). Each row of the table represents one type of speech problem and shows the number of syndromes associated with it, the mean and median of the counts of chainlink genes for the syndromes, and the mean and median of the chainlink connectivities of the syndromes. From an inspection of Table 2, a distinct pattern emerges. Rank ordering the symptoms by ascending order of the means of the counts of chainlink genes, we see that their connectivities also fall in almost the same order:

(1) $$\begin{array}{c} \mathrm{normal}(32,404)<\mathrm{apraxia}(43,675)<\mathrm{dysarthria}(43,725)<\mathrm{impaired}(46,734)\\ <\mathrm{delayed}(47,682)<\mathrm{absent}(60,902) \end{array}$$

This rank ordering is consistent with the rank ordering of symptom severity based on the descriptions in the medical literature. In general, statistically speaking, the *number* of chainlink genes and the chainlink connectivity both appear to relate monotonically to the severity of the speech disorder.

**Figure 3:**
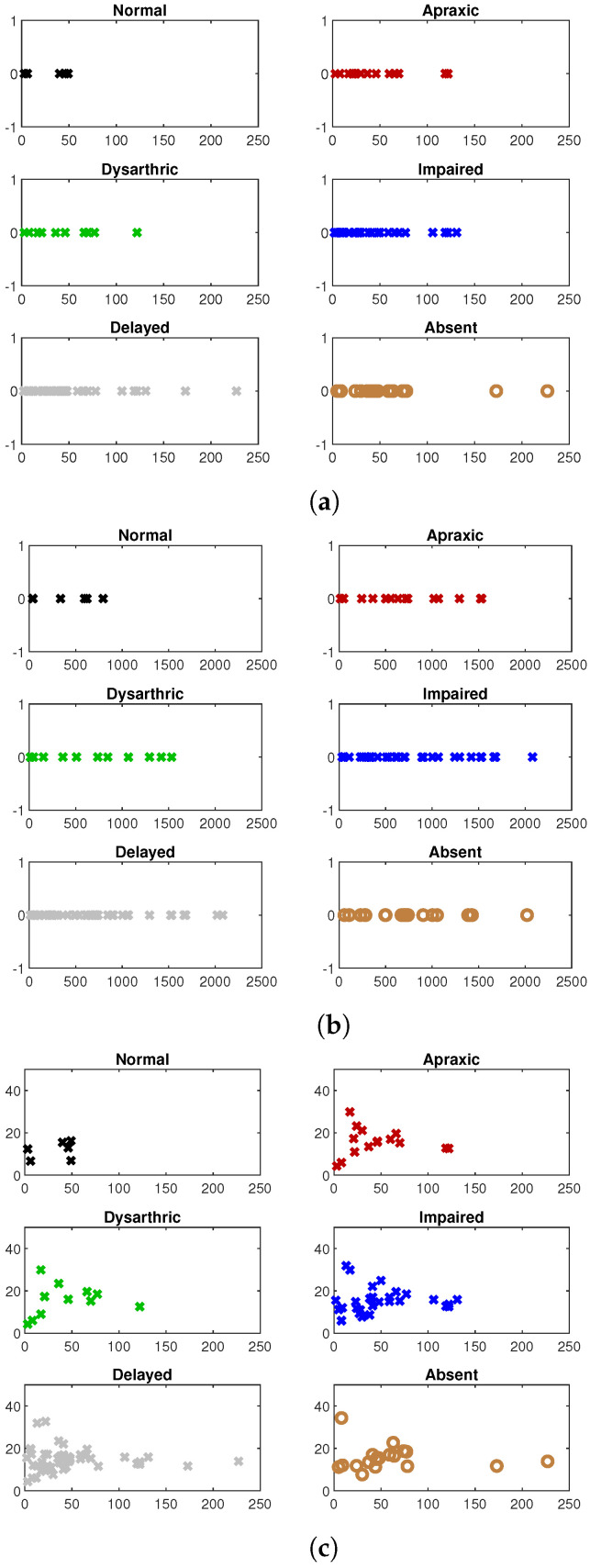
(**a**) **Chainlink counts**: Scatter of chainlink gene counts for syndromes associated with voice problems of different severities. Each cross represents a single syndrome. The horizontal axis value indicates the number of chainlink genes for the syndrome. The *y*-axis is a dummy axis. (**b**) **Chainlink connectivity**: Scatter of chainlink connectivities for syndromes associated with voice problems of different severities. Each panel shows the aggregate no. of pathways that all chainlink genes connect to (*x*-axis) for each syndrome (denoted by a cross) that exhibits the labeled speech characteristic. The *y*-axis is a dummy axis. (**c**) **Count vs. normalized connectivity**: Chainlink gene count (*x*-axis) vs. normalized chainlink gene connectivity (*y*-axis). The normalized connectivity is the average connectivity per chainlink gene.

Figure 3 shows scatter plots for counts of chainlink genes, chainlink connectivity, and a scatter of chainlink gene counts vs. normalized (per-chainlink-gene) chainlink connectivity for different severities of voice problems. Once again, it is apparent from the figures that the distributions of chainlink counts and chainlink connectivity is predictive of the type of speech problem. In particular, as is evident from Figure 3c, the distribution for normal speech stands out distinctly, as does that for absent speech, although the latter is not as distinctive as the former. Among the other levels, the distributions for apraxic and dysarthric speech appear similar, and so also do those for impaired and delayed speech appear similar.

In order to quantify these differences, we modeled the distributions of chainlink counts and chainlink connectivities for the different severity levels. These distributions have the general characteristics of over-dispersed Poisson distributions and can be modelled as Conway–Maxwell–Poisson (CMP) distributions [67]. The CMP distribution is a two-parameter exponential-family PMF over non-negative integers that takes the form

(2) $$P\left(n\right)=\frac{\lambda^{n}}{{\left(n!\right)}^{\nu }}\frac{1}{Z(\lambda ,\nu )}$$

where λ,ν>0 are the parameters of the distribution. Given a set of integers, λ and ν can be obtained through a maximum likelihood estimator [68].

**Figure 4:**
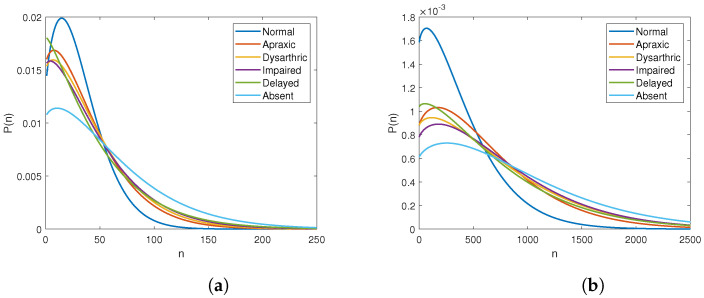
Conway–Maxwell–Poisson models for the distributions of chainlink counts and chainlink connectivities for different types of speech problems. (**a**) Distribution of chainlink counts. (**b**) Distribution of chainlink connectivities.

Figure 4 shows the maximum likelihood estimates of the probability distributions of chainlink counts (Figure 4a) and connectivities (Figure 4b) for syndromes associated with speech problems of different severity levels. These, too, follow the visible trends of Figure 3, where the distributions of both chainlink counts and chainlink connectivities for syndromes associated with the two extreme conditions, normal and absent speech, are distinct from those for other types of problems.

To quantify the differences in the distributions, we define the *code distance* between two sets of integers $C_{i}=\left\{n_{1},\cdots ,n_{i}\right\}$ and $C_{j}=\left\{m_{1},\cdots ,m_{j}\right\}$ as the excess number of bits required to encode them if each set is encoded using the optimal code for the other set rather than itself.

(3) $$D\left(P_{i},P_{j}\right)=\sum_{n\in C_{i}}\log_{2}\left(\frac{P_{j}\left(n\right)}{P_{i}\left(n\right)}\right)+\sum_{n\in C_{j}}\log_{2}\left(\frac{P_{i}\left(n\right)}{P_{j}\left(n\right)}\right)$$

where $P_{i}\left(\right)$ and $P_{j}\left(\right)$ are the estimates of the PMFs for $C_{i}$ and $C_{j}$, respectively. In our case, we choose the maximum likelihood estimates of the CMP distributions for the sets to compute this metric.

Table 3a shows the code distances between the chainlink counts for different types of speech problems. Table 3b shows the same for their chainlink connectivities. In both cases, we observe that the distributions for normal speech stand clearly apart from those for the other types of speech problems. The distributions for fully absent speech, too, are distinctive from those for other problem types. Among apraxic, dysarthric, impaired, and delayed speech, the differences between the distributions of adjacent degrees of severity is minimal; however, the distances show a distinct increasing trend with increases in the degree of impairment.

Overall, from the above analysis, the properties of the level-2 chains of a syndrome appear predictive of the degree of the speech problems associated with it. Our analysis has considered the chainlink counts and connectivities indpendently, and each of them shows this behavior. A joint analysis of both may show stronger dependencies.

Most importantly, note that in all of the analysis above, we have ignored the secondary effects of other issues, such as intellectual disability and craniofacial anomalies, a highly simplifying assumption. A more correct information measure that takes these into account is expected to show even stronger relationships between the level and degree of connectivity of a syndrome to the FOXP2 pathway and its effect on speech.

### 4.3. Why Are There No Instances of Missing Voice Chains?

Are voice chains redundant? The fact that there are no missing voice chains is easily explainable. The reason is linked to the size of the syndromic regions. To understand this, consider the following facts.

Our database comprises 4319 unique pathways. A total of 1205 of these pathways are linked to the pathway that carries the FOXP2 gene, and collectively, these include 11,746 genes. Thus, a randomly chosen gene from the entire human genome of 42.7k genes (as in the HGNC Human Genome database) has a 27.5% chance of being on a pathway that links to the FOXP2 containing pathway, i.e., of being a level-2 chainlink gene.

The shortest microdeletion considered (2q23.1) includes 9 genes, each of which has a 27% chance of being a level-2 chainlink gene. The syndrome itself then has a 94.44% probability of having a level-2 voice chain purely by chance. The second shortest pathway includes 26 genes and has a 99.98% probability of having a level-2 voice chain by chance. The remaining pathways are larger (in terms of the number of genes), and it is virtually impossible for them *not* to have a level-2 chain.

As a result, it is realistic to expect that, as a consequence of the density with which the FOXP2 containing pathway is linked to other pathways, *any syndrome arising from genetic aberrations that includes even a moderately sized set of genes will have an effect on voice*. It remains a plausible hypothesis that any factor that influences gene function has at least some chance to ultimately affect voice—for example, at least a 27% chance within the boundaries of the example presented in this paper.

The above argument assumes that the genes in a microdeletion region are randomly chosen. The mean of the fraction of genes in a microdeletion that appears in any voice chain is observed to be 28.79% with a variance of 0.014, indicating concordance with the assumption of randomness. A secondary implication is that the likelihood of adjacent genes in the same cytogenetic region to be chainlink genes is independent of one another.

### 4.4. Ancillary Observations

Some important ancillary observations emerge from this study, which may be important to note. These are mentioned briefly below.

1. For each syndrome, some genes have been identified as largely important—i.e., these are *implicated* largely for the syndrome’s effect on the individual. Of the syndromes for which there is information about implicated genes, we see that in only 8 syndromes (2p16.1–p15, 2q23.1, 9p24.3, 11q23, 13q12.3, 17q23.1–q23.2, 19p13.13, and Yq11), none of the implicated genes appear in the two levels of voice chains shown. In all other cases, the implicated genes impact FOXP2 pathways and are likely to have a bearing on speech anomalies. We have noted earlier that FOXP2 is not the only gene known to be related to voice production. If we had chosen some other gene as an example in this paper (instead of FOXP2), it is likely that the implicated genes for the 8 exceptions mentioned above would appear as chainlink genes (while some others may not). This hypothesis can be easily tested in corresponding experiments.
2. **Identifying candidate genes for further investigation:** Using only chainlink genes that appear on level-1 chains as illustrative examples (see Table A1 in the Appendix A for reference), we see that voice chains can be useful in identifying candidate genes for further investigation in the context of speech issues. Some examples are given below. The likely candidates are written in parentheses, while the already implicated genes are indicated in bold:
  - 1p36 (ARID1A): Although not implicated for this syndrome in studies so far, ARID1A is located in 1p36.11, a region frequently deleted in human cancers [69]. Disruption in its function may lead to the co-occurrence of oncological and speech issues. This hypothesis is verifiable.
  - 5q35.3 (**NSD1**): The gene NSD1 appears in a level-1 chain and is also an implicated gene. Ideally, this should not be a candidate for further investigation. However, paradoxically, while effects on speech are expected, the literature reports normal speech for some subjects for this case. This may be a result of biased sampling (the more severe cases may not be conducive to life due to other concurrent severe symptoms, which is a common occurrence in microdeletion syndromes; in some cases, only mosaic individuals survive). This warrants some investigation.
  - 11p15.5 (HRAS): Although not implicated, and although two studies cited under OMIM: 130650 for this syndrome explicitly mention HRAS as *not* significant, HRAS has nevertheless been independently found to be extremely significant in RASopathy and cancer studies, e.g., [70]. Its role in this syndrome needs to be re-evaluated given its influence on 347 biological pathways and its strong influence on speech.
  - 16p11.2 and 16p12.2–16p11.2 (SRCAP): Although not implicated, it connects to only one other pathway in the ensemble, and that is the ACC pathway of FOXP2. The effects on speech are expected to be strong if this gene is aberrant. This gene may be implicated in further investigations.
  - 17p13.1 (KDM6B): Speech is absent in this syndrome. The gene TP53 is implicated, which also appears at level-1 and is associated with 206 pathways. KDM6B is the only other gene in the level-1 voice chains and connects to only 8 other pathways. It is likely that this gene also plays a strong role in influencing speech and merits investigation.
  - 17q12 (ERBB2): The gene ERBB2 is associated with 124 pathways. It is a well-known oncogene [71], in that perturbations in its function have been observed to have deleterious effects. If it is also connected to FOXP2, then its appearance in the voice chain allows a surprising hypothesis—that biomarkers of some oncological conditions may also be present in voice.
  - 19p13.3 (MAP2K2,UHRF1): MAP2K2 and URHF1 are not implicated. However their appearance as level-1 chainlink genes warrants investigation, especially for MAP2K2, which influences 257 pathways. Prompted by this, a literature search did reveal that MAP2K2 *has* been implicated in this syndrome recently [72], although this is not on the OMIM records, which were largely consulted for this study.
  - 22q13.3 (BRD1): The gene BRD1 is not implicated and appears in 9 pathways only, but the effect on speech is severe in this syndrome. This warrants the investigation of BRD1 independently in relation to speech characteristics. A literature search reveals that BRD1 is indeed strongly associated with brain development and susceptibility to both schizophrenia and bipolar affective disorder [73], and consequent effects on speech are highly likely.
  - Xp11.22 (SMC1A): Although SMC1A is not implicated, it appears in 33 pathways. The speech issues are severe and the gene warrants investigation for this effect. A recent report in the literature has implicated it in severe intellectual disability and therapy-resistant epilepsy in females [74]. The former is known to be associated with severe speech anomalies.
  - Xp11.3 (KDM6A): Although not implicated, KDM6A warrants investigation. In the literature, it is independently known to be associated with delayed speech and psychomotor development [75].
3. **Expression of speech characteristics:** The observation that deletions of genes on *all chromosomes* ultimately results in the expression of speech anomalies carries significance. From a much broader perspective, this suggests that the effect on speech may be supported by the action of multiple concurrent biological pathways. There may be no single gene or genes (on select chromosomes) that may code for speech capabilities per se, and FOXP2 may be one of a few genes that may *consolidate and regulate* the speech- and language-related emergent effects. It may be that genes directly code for structural elements in the range of phenotypes, while other properties, such as speech and language abilities, are emergent from the coordination of these (and epigenetic) factors.
  A more prosaic argument for this can also be presented. Within the ensemble of syndromes analyzed, there are three kinds of of cause-and-effect relationships: (a) syndromes with *physical* structures of the vocal tract (e.g., craniofacial anomalies that include cleft palate, changes in lip shape, etc.), which adversely affect the biomechanical aspects of voice and speech production, (b) syndromes in which auditory and motor functions are compromised, and (c) syndromes that affect the normal functions of the brain, causing cognitive, learning, memory, and other issues that are, in turn, likely to lead to speech problems. In no case do we see only speech aberrations in isolation of these factors. The associations between speech and other expressed factors have, in fact, been ubiquitously observed, e.g., [48]. This may support the hypothesis that speech abilities are likely to be emergent from an ensemble of factors (including other phenotypes), rather than expressed directly by any “speech” gene.

## 5. Conclusions

The hypothesis that the existence of voice chains is correlated with speech characteristics is adequately validated by the statistical analysis presented in this paper. The analysis presented in this paper, in fact, also shows that the level of voice chains is correlated positively with the severity of speech problems. Based on this, a simple information measure has been suggested to rank-order the effects of specific sets of voice chains on speech. We also see how the methodology presented can potentially provide leads to specific genes that might be candidates for further investigation in the context of speech issues and microdeletion syndromes. While the example of chromosomal microdeletion syndromes used for this paper is very specific, the methodology itself may be easily generalized and extended to reveal the potential effects of other diseases with a genetic basis and of other factors that influence gene function in some manner on speech, voice and (in further refinements of the analysis) their specific qualities and characterisitcs. As a specific suggestion, one exercise that would allow for a more comprehensive analysis would be to explore the entire human genome database to identify which genes are connected via voice chains (and to what level), as well as whether or not there have been corresponding effects on voice reported in the biomedical literature. In cases where large amounts of data are available, one could also explore such connections in an entirely data-driven manner, using AI-based biomarker discovery mechanisms.

## Figures and Tables

**Table 1:** Information about level-1 and level-2 voice chain ensembles for 76 chromosomal microdeletion syndromes. For each syndrome, the corresponding implicated gene (culled from medical literature, with details given in the Appendix A) that was *also discovered to be a chainlink gene by the proposed algorithm* in either level-1 or level-2 chains is listed in the second column. The number of chainlink genes (i.e., genes that have chains that connect to the ACC biological pathway of FOXP2) is shown in the third and fourth columns for level-1 and level-2 chains, respectively. The total number of pathways that they collectively influence is indicated next to each count, in parentheses. The observed phenotypic effects on speech are given in the last column. **Del:** delayed speech; **Imp:** impaired speech; **Norm:** normal speech; **Abs:** absent speech; **Apr:** apraxia; **Dys:** dysarthria; **Idio:** idiosyncratic.

Syndrome	Implicated Genes from Microarray Studies That Also form Voice Chains	No. of Genes in $\left\{V_{N1}\right\}$	No. of Genes in $\left\{V_{N2}\right\}$	Reported Effects on Speech
1p36	SPEN	1 (10)	226 (3152)	Del [9], or Abs
1q21.1–q21.2	RBM8A, GJAS	–	41 (906)	Del [10] or Imp [11]
1q41–q42	DISP1, LEFTY1, LEFTY2, BPNT1	–	60 (1020)	Apr [12]
1q43–q44	AKT3	–	78 (903)	Del, Imp, or Abs
2p16.1–p15		–	17 (508)	Dys, Apr, or Imp [13]
2p21	SLC3A1	–	24 (557)	Apr or Idio [14]
2q23.1		–	2 (31)	Del or Imp
2q32–q33	COL3A1, COL5A2, GTF3C3, CASP8, CASP10	–	62 (1429)	Abs
2q37.3	HDAC4	–	38 (330)	Imp [15]
3p13	FOXP1	–	8 (48)	Del, Idio, Imp and Dys (all severe), Apr [16]
3q13.31	DRD3, GAP43, LSAMP	–	5 (56)	Imp [17] or Abs
3q29	PAK2, DLG1	–	24 (413)	Del
4p16.3	FGFR3	1 (25)	44 (495)	Del [18] or Abs
4q21		–	41 (696)	Del or Abs; Imp [19,20]
5p (5p15.2 and/or (5p15.3 or 5p15.33))	TERT, CTNND2	–	37 (500)	Del, Abs, and Apr [21]
5q14.3	MEF2C	–	7 (275)	Abs
5q33.1	RPS14	–	17 (153)	Dys [22]
5q35.3	NSD1	1 (8)	47 (653)	Norm [23] or Del
6pter–p24	FOXC1, GMDS	–	41 (416)	Del
6q25.3	ARID1B	–	21 (363)	Del, Apr, Dys [24]
7p21	TWIST1	–	18 (243)	Del [25]
7q11.23	ELN, LIMK1, GTF2IRD1, GTF2I	–	35 (536)	Norm or Del
8p23.1	GATA4	–	49 (337)	No significant anomaly reports
8q22.1	CCNE2	–	13 (152)	Del
8q24.11–q24.13	EXT1	–	21 (211)	Del
9p24.3		1 (9)	3 (13)	Del, Dys, Apr [26]
9q34.3	EHMT1	1 (62)	46 (718)	Del [27], Apr [28] and Abs
10pter–p13 or 10p14–p15.1	GATA3	–	49 (796)	Sensorineural hearing loss
10q23	PTEN, BMPR1A	1 (107)	59 (1003)	Del or Abs; Imp [29]
10q26	DOCK1	–	48 (708)	Imp; Del [30]
11p11.2–p12	EXT2	–	46 (737)	Idio, Dys, Del, Apr [31]
11p13–p12	PAX6, SLC1A2, PRRG4	–	23 (342)	Imp [32]
11p15.5	IGF2	1 (347)	50 (1243)	Imp [33]
11q13.3	FGF4, FGF3, FADD	–	11 (608)	Del [34] Imp [35]
11q23		–	77 (1421)	Dysarthric, Abs [36]; Imp [37]
11q23.3-q25	FLI1, JAM3	–	119 (1522)	Imp, Del, Apr [38]
12q14.3	HMGA2	–	9 (108)	Abs, Imp [38] or Del [39]
13q12.3		–	6 (119)	Del [40]
13q14	RB1	–	46 (595)	Norm [41]
13q22.3	EDNRB	–	3 (37)	Norm [41]
13q33–q34	SOX1, ARHGEF7	–	30 (635)	Apr
14q11–q22	PAX9, SUPT16H, CHD8, RALGAPA1	–	173 (2021)	Del [42] or Abs
14q22.1–q23.1	PTGDR, BMP4	–	41 (530)	Imp [43]
14q32.2	DLK1, MEG3	–	23 (242)	Del; Idio [44]
15q11.2	NIPA1, NIPA2, CYFIP1, TUBGCP5	–	11 (72)	Del
15q11–q13	NDN, SNRPN	–	30 (230)	Del or Imp
15q11–q13	UBE3A		–Same as above–	Abs; Imp [45]
15q13.3	CHRNA7, OTUD7A	–	8 (47)	Imp or Idio
15q24	SIN3A	–	38 (623)	Del or Imp
16p11.2	SH2B1, TBX6, CORO1A	1 (1)	70 (1067)	Apr [46]; Dys [47], Del or Imp
16p12.2–p11.2	SH2B1	1 (1)	106 (1682)	Del or Imp
16p12.1		–	17 (157)	Del
16p13.11	MYH11	–	6 (116)	Del [48]
16p13.3	CREBBP, TRAP1	1 (140)	122 (1532)	Apr, Dys, Imp or Del [49]
16q22	CBFB	1 (2)	79 (830)	–Not available–
16q24.3–q24.2	CDH15, ZNF778, ZFPM1	–	28 (307)	Del or Imp [50]
17p11.2	LLGL1, UBB	–	36 (845)	Del; Dys [51]
17p13.1	KCNAB3, GUCY2D, TP53, TRAPPC1, MPDU1, FXR2, EFNB3	2 (214)	74 (1387)	Abs
17p13.3	PAFAH1B1, YWHAE	–	37 (487)	Del [52]
17q11.2	NF1	–	40 (620)	No significant issues
17q12	HNF1B, LHX1, CCL3L3	1 (124)	59 (890)	Del or Imp
17q21.31	KANSL1, MAPT, CRHR1	2 (68)	46 (740)	Del or Abs
17q23.1–q23.2		–	28 (307)	Del [53]
17q24.3–q24.2	ABCA5, MAP2K6, SOX9	–	22 (720)	Del [54]
18q	MBP	–	122 (1666)	Del [55] or Imp [56]
19p13.13		2 (263)	131 (2080)	Del; Imp [57]
19q13.11	UBA2, WTIP	1 (23)	22 (283)	Del or Abs; Imp [58]
20p12.3	BMP2	–	13 (415)	Del or Imp [59]
22q11.2	TBX1, COMT, TOP3B	–	66 (1294)	Apr, Dys, Del or Imp [60]
22q12.2	NF2	–	27 (253)	Del or Imp [61]
22q13.3	ARSA, SHANK3	1 (9)	45 (670)	Del or Abs
Xp11.3	RP2	1 (6)	20 (335)	Imp [62]
Xp21	GK, DMD, NR0B1	–	7 (121)	Del [63]
Xq28 (a)	ABCD1, BCAP31, SLC6A8	–	64 (1053)	Del [64]
Xq28 (b)	MECP2	–	–same as above–	Norm to Abs
Yq11		–	6 (40)	Norm

**Table 2:** **Row-wise:** Statistics showing the number of syndromes (count) associated with each voice disorder, the number of chainlink genes associated with the corresponding set of syndromes, and the connectivity of chainlink genes for the set.

Speech Type	Count	Chainlink Genes		Chainlink Connectivity	
		Mean	Median	Mean	Median
Normal	6	32	40	404	595
Apraxic	19	43	30	675	557
Dysarthric	11	43	36	725	737
Impaired	31	46	38	734	608
Delayed	51	47	37	682	500
Absent	19	60	46	902	718

**Table 3:** (**a**) Code distance between the (sets of) chainlink counts for different types of speech disorders. (**b**) Code distance between the (sets of) chainlink connectivities for diferrent types of speech disorders.

(a)						
	**Normal**	**Apraxic**	**Dysarthric**	**Impaired**	**Delayed**	**Absent**
Normal	0	2.1	2.3	7.6	14.7	15.3
Apraxic		0	0.2	1.1	2.9	6.8
Dysarthric			0	0.2	1.2	3.7
Impaired				0	0.5	3.7
Delayed					0	4.6
Absent						0
(**b**)						
	**Normal**	**Apraxic**	**Dysarthric**	**Impaired**	**Delayed**	**Absent**
Normal	0	6.3	7.0	17.4	22.5	22.2
Apraxic		0	0.7	1.5	1.9	5.9
Dysarthric			0	0.0	0.4	1.7
Impaired				0	0.9	2.4
Delayed					0	5.5
Absent						0

## Data Availability

The code required to reproduce the results in this paper is archived for public use at https://datadryad.org/ (accessed on 21 September 2022) under the title “Data for Connecting voice profiling to genomics”.

## Appendix A

This table lists the voice chains found for a set of 76 documented microdeletion syndromes. This range excludes chromosome 21, for which sufficient documentation was not found in the literature. The analysis was conducted for voice chains up to level 2. The format of each row in this table is as follows:

**First column:**

- In each row, the top left entry is the microdeletion syndrome.
- Below this, on the left, is the OMIM record for the cytogenetic region of the syndrome.
- Below the OMOM record, the common names by which the syndrome is referred to in the medical literature are listed.
- Below the list of common names, sets of chainlink genes that form level-1 and level-2 voice chains (denoted as $\left\{V_{N1}\right\}$ and $\left\{V_{N2}\right\}$) respectively, found by the proposed algorithm are listed. These sets are listed only if they are present.
- For each voice chain listed, the first entry denotes the level of the voice chain. Following this is the list of chainlink genes that belong to the corresponding microdeletion region, which are linked to FOXP2 through the corresponding voice chain level.
- For each gene, the number of pathways that a gene connects to (in general, inclusive of connections to the ACC pathway of FOXP2) is written as its subscript.
- All chainlink genes are not named. For brevity, the first 10 genes with the greatest number of links are listed, and the total number of the rest of the genes (total chainlink gene count) is indicated. At the end, the genes with a single pathway link are explicitly listed. They are *included* in the total chainlink gene count mentioned above. Thus, for example, the level-2 voicechain for the syndrome 2p16.1–p15 has a total chainlink gene count of 17, *including* all genes that are explicitly listed in the table.
- In each row, the genes in the voice chains that have been implicated for the syndrome’s phenotypic expression in prior studies are shown in parentheses on the top right. The genes that are also present as chainlink genes in the voicechains found for the syndrome are shown in bold font.

**Second column:**

- The second column in this table lists the corresponding speech characteristics, with references. Where no reference is cited, the information is found in the OMIM record for the syndrome (where possible, OMIM references are used for brevity).

**Table A1:** Chainlink genes for level-1 and level-2 voice chain ensembles for 76 chromosomal microdeletion syndromes. Each chain forms a link from the gene shown to the ACC biological pathway of FOXP2 and has been automatically derived. The total number of pathways that each gene influences independently is shown as a subscript to its name. Observed phenotypic effects on speech are given in the last column. When references are not cited, the information reflects that in the OMIM records for the syndrome.

Syndrome	(Implicated Genes from Microarray Studies)	Reported Effects on Speech
Other Information		
Voice Chains and Their Member Chainlink Genes		
**Chromosome 1 deletions**		
**1p36**	(Multiple incl. RERE, **SPEN**) ${}^{†‡}$	Delayed [9] or absent speech
**OMIM:** 607872; Cytogenetic location: 1p36; Genomic coordinates (GRCh38): 1:23,600,000–27,600,000 **Names:** Chromosome 1p36 deletion sydrome; monosomy 1p36 syndrome $\left\{V_{N1}\right\}$ ARID1A${}_{10}$ $\left\{V_{N2}\right\}$: PIK3CD${}_{168}$ MTOR${}_{146}$ GNB1${}_{140}$ CDC42${}_{140}$ CASP9${}_{108}$ RPS6KA1${}_{93}$ PRKCZ${}_{75}$ CNKSR1${}_{57}$ SFN${}_{55}$ DVL1${}_{48}$ (216more…) SSU72${}_{1}$ RBP7${}_{1}$ PLEKHN1${}_{1}$ PLEKHM2${}_{1}$ PEX10${}_{1}$ NBL1${}_{1}$ MTCO3P12${}_{1}$ MMP23B${}_{1}$ MMP23A${}_{1}$ HSPB7${}_{1}$ HMGN2${}_{1}$ GPR3${}_{1}$ EXOSC10${}_{1}$ DISP3${}_{1}$ CROCCP2${}_{1}$ CAMTA1${}_{1}$ AHDC1${}_{1}$ ACAP3${}_{1}$		
1q21.1–q21.2	(Multiple incl. **RBM8A** ${}^{†‡}$, **GJAS** ${}^{†}$)	Delayed [10] or Impaired [11] speech
**OMIM:** 612474/274000, Cytogenetic location: 1q21.1 Genomic coordinates (GRCh38): 1:143,200,000–147,500,000 **Names:** Thrombocytopenia with absent radius (TAR) syndrome (OMIM 274000) $\left\{V_{N2}\right\}$: H4C14${}_{67}$ H4C15${}_{66}$ H2BC21${}_{63}$ PRKAB2${}_{57}$ H3C15${}_{56}$ H3C14${}_{56}$ H3C13${}_{56}$ H2AC20${}_{53}$ H2AC19${}_{53}$ H2AC18${}_{53}$ (31more…)		
1q41–q42	(Multiple incl. **DISP1**, HPE10, **LEFTY1**, **LEFTY2**, WDR26, TSEN2, **BPNT1**) ${}^{†‡}$	Apraxia [12]
**OMIM:** 612530; Cytogenetic location: 1q41–q42 Genomic coordinates (GRCh38): 1:214,400,000–236,400,000 **Names:** Chromosome 1q41–q42 deletion syndrome; holoprosencephaly 10; HPE10 $\left\{V_{N2}\right\}$: NUP133${}_{88}$ H2BU1${}_{63}$ TGFB2${}_{62}$ DUSP10${}_{54}$ H3-3A${}_{50}$ WNT3A${}_{47}$ ARF1${}_{46}$ PARP1${}_{41}$ MIR3620${}_{38}$ PSEN2${}_{36}$ (50more…) **DISP1**${}_{2}$ MIR215${}_{1}$ MIR194-1${}_{1}$ DNAH14${}_{1}$ CDC42BPA${}_{1}$		
1q43–q44	(**AKT3**, ZBTB18) ${}^{‡}$	Delayed, impaired, or absent speech
**OMIM:** 612337 **Names:** Mental retardation, autosomal dominant 22; MRD22; chromosome 1q43–q44 deletion syndrome (included); chromosome 1qter deletion syndrome (included) $\left\{V_{N2}\right\}$: **AKT3**${}_{169}$ RYR2${}_{74}$ ACTN2${}_{69}$ CHRM3${}_{42}$ MTR${}_{38}$ FH${}_{31}$ ADSS2${}_{30}$ EXO1${}_{26}$ RGS7${}_{16}$ KMO${}_{15}$ (68more…) FMN2${}_{1}$		
**Chromosome 2 deletions**		
2p16.1–p15	(Multiple incl. BCL11A) ${}^{†‡}$	Dysarthria, apraxia, or impaired speech [13]
**OMIM:** 612513; Cytogenetic location: 2p16.1–p15 Genomic coordinates (GRCh38): 2:54,700,000–63,900,000 **Names:** Chromosome 2p16.1–p15 deletion syndrome $\left\{V_{N2}\right\}$: RPS27A${}_{293}$ XPO1${}_{56}$ UGP2${}_{33}$ MDH1${}_{27}$ REL${}_{23}$ CCT4${}_{14}$ RTN4${}_{13}$ B3GNT2${}_{12}$ VRK2${}_{10}$ USP34${}_{7}$ (7more…) OTX1${}_{1}$ CCDC88A${}_{1}$		
2p21	(Multiple incl. **SLC3A1**, PREPL)	Apraxia or idiosyncratic speech [14]
**OMIM:** 606407; Cytogenetic location: 2p21 Genomic coordinates (GRCh38): 2:41,500,000–47,500,000 **Names:** Hypotonia–cystinuria syndrome; cystinuria with mitochondrial disease; homozygous 2p21 deletion syndrome $\left\{V_{N2}\right\}$: CALM2${}_{234}$ PRKCE${}_{73}$ **SLC3A1**${}_{36}$ HAAO${}_{22}$ ATP6V1E2${}_{19}$ ABCG5${}_{18}$ MSH2${}_{17}$ ABCG8${}_{17}$ EPAS1${}_{16}$ COX7A2L${}_{15}$ (14more…)SIX3${}_{1}$ EML4${}_{1}$		
2q23.1	(MBD5) ${}^{‡}$	Delayed or impaired speech
**OMIM:** 156200 **Names:** Mental retardation, autosomal dominant 1; MRD1; chromosome 2q23.1 deletion syndrome $\left\{V_{N2}\right\}$: ORC4${}_{29}$ KIF5C${}_{2}$		
2q32–q33	(SATB2) ${}^{‡}$(HOXD cluster and regulatory elements, **COL3A1 COL5A2**, **GTF3C3**, **CASP8**, **CASP10**) ${}^{‡‡}$	Absent speech
**OMIM:** 612313 **Names:** Glass syndrome; GLASS; chromosome 2q32–q33 deletion syndrome; SATB2-associated syndrome $\left\{V_{N2}\right\}$: CREB1${}_{203}$ STAT1${}_{130}$ **CASP8**${}_{103}$ NUP35${}_{79}$ ITGAV${}_{64}$ CD28${}_{60}$ SUMO1${}_{52}$ AOX1${}_{50}$ FZD5${}_{43}$ FZD7${}_{37}$ (52more…) TMEFF2${}_{1}$ KLF7${}_{1}$ HSPE1${}_{1}$ DUSP19${}_{1}$ DNAH7${}_{1}$		
2q37.3	(**HDAC4**) ${}^{‡}$	Impaired speech [15]
**OMIM:** 600430 (2q37); Cytogenetic location: 2q37 Genomic coordinates (GRCh38): 2:236,400,000–242,193,529 **Names:** Chromosome 2q37 deletion syndrome, brachydactyly–intellectual disability syndrome; Albright hereditary osteodystrophy-like syndrome Type 3 $\left\{V_{N2}\right\}$: **HDAC4**${}_{33}$ GPC1${}_{27}$ AGXT${}_{25}$ COL6A3${}_{19}$ ACKR3${}_{14}$ NEU4${}_{13}$ NDUFA10${}_{12}$ PRLH${}_{11}$ PER2${}_{11}$ DTYMK${}_{11}$ (28more…)TWIST2${}_{1}$ ILKAP${}_{1}$		
**Chromosome 3 deletions**		
3p13	(**FOXP1**) ${}^{†‡}$	Delayed, idiosyncratic, and impaired speech and dysarthria (all severe), apraxia [16]
**OMIM:** 613670 **Names:** Mental retardation with language impairment with or without autistic features $\left\{V_{N2}\right\}$: MITF${}_{19}$ PROK2${}_{8}$ PPP4R2${}_{5}$ GPR27${}_{5}$ EIF4E3${}_{5}$ **FOXP1**${}_{3}$ GXYLT2${}_{2}$ RYBP${}_{1}$		
3q13.31	(**DRD3**, ZBTB20 ${}^{‡}$, **GAP43**, **LSAMP**) ${}^{†‡}$	Impaired [17] or absent speech
**OMIM:** 615433; Cytogenetic location: 3q13.31 Genomic coordinates (GRCh38): 3:113,700,000–117,600,000 **Names:** Chromosome 3q13.31 deletion syndrome $\left\{V_{N2}\right\}$: **DRD3**${}_{22}$ ATP6V1A${}_{22}$ **GAP43**${}_{6}$ QTRT2${}_{3}$ **LSAMP**${}_{3}$		
3q29	(Multiple incl. **PAK2**, **DLG1**) ${}^{†‡}$	Delayed speech
**OMIM:** 609425; Cytogenetic location: 3q29 Genomic coordinates (GRCh38): 3:192,600,000–198,295,559 **Names:** Chromosome 3q29 deletion syndrome; microdeletion 3q29 syndrome; 3qter deletion syndrome $\left\{V_{N2}\right\}$: **PAK2**${}_{74}$ **DLG1**${}_{71}$ NCBP2${}_{53}$ HES1${}_{39}$ RPL35A${}_{22}$ TFRC${}_{21}$ RNF168${}_{18}$ BDH1${}_{17}$ PCYT1A${}_{15}$ MUC20${}_{13}$ (14more…)FBXO45${}_{1}$		
**Chromosome 4 deletions**		
4p16.3	(Multiple incl. **FGFR3**, MSX1) ${}^{†‡}$	Delayed [18] or absent speech
**OMIM:** 194190; Cytogenetic location: 4p16.3 Genomic coordinates (GRCh38): 4:0–4,500,000 **Names:** Wolf–Hirschhorn syndrome; Pitt–Rogers–Danks syndrome; Pitt syndrome; Wittwer syndrome; Dillan 4p syndrome $\left\{V_{N1}\right\}$ CTBP1${}_{25}$ $\left\{V_{N2}\right\}$: **FGFR3**${}_{109}$ TNIP2${}_{32}$ NELFA${}_{27}$ MIR943${}_{26}$ CTBP1${}_{25}$ ADRA2C${}_{22}$ NSD2${}_{19}$ GRK4${}_{15}$ DGKQ${}_{14}$ PDE6B${}_{13}$ HAUS3${}_{13}$ SLC26A1${}_{12}$ SLBP${}_{12}$ ATP5ME${}_{12}$ RNF4${}_{11}$ CPLX1${}_{11}$ RGS12${}_{10}$ (27more…) ZFYVE28${}_{1}$ PCGF3${}_{1}$ NSG1${}_{1}$ MXD4${}_{1}$ ABCA11P${}_{1}$		
4q21	(Multiple)	Delayed or absent speech; impaired speech [19,20]
**OMIM:** 613509; Cytogenetic location: 4q21 Genomic coordinates (GRCh38): 4:86,000,000–87,100,000 **Names:** Chromosome 4q21 deletion syndrome $\left\{V_{N2}\right\}$: MAPK10${}_{122}$ FGF5${}_{118}$ NUP54${}_{79}$ PAQR3${}_{47}$ PRKG2${}_{27}$ CXCL10${}_{22}$ CXCL9${}_{18}$ ABRAXAS1${}_{18}$ SEC31A${}_{16}$ CXCL11${}_{16}$ (31more…) SHROOM3${}_{1}$ SEPTIN11${}_{1}$ HNRNPDL${}_{1}$ G3BP2${}_{1}$ BMP2K${}_{1}$ AFF1${}_{1}$		
**Chromosome 5 deletions**		
5p (5p15.2 and/or (5p15.3 or 5p15.33))	(Multiple incl. **TERT**, **CTNND2**) ${}^{†}$	Delayed or absent speech and apraxia [21]
**OMIM:** 123450 **Names:** Chromosome 5p deletion syndrome; cri-du-chat syndrome; cat cry syndrome; Lejeune syndrome; 5p monosomy; partial monosomy 5 $\left\{V_{N2}\right\}$: ADCY2${}_{116}$ SLC6A3${}_{73}$ SDHA${}_{37}$ **TERT**${}_{24}$ TRIO${}_{23}$ MTRR${}_{19}$ LPCAT1${}_{15}$ CEP72${}_{15}$ SRD5A1${}_{14}$ SLC9A3${}_{13}$ (27more…)OTULINL${}_{1}$ **CTNND2**${}_{1}$ CLPTM1L${}_{1}$		
5q14.3	(**MEF2C**) ${}^{‡}$	Absent speech
**OMIM:** 612881 (distal version 5q14.3–q15); Cytogenetic location: 5q14.3–q15 Genomic coordinates (GRCh38): 5:83,500,000–98,900,000 **Names:** Distal chromosome 5q14.3 deletion syndrome; periventricular heterotopia associated with chromosome 5q deletion; periventricular nodular heterotopia 5; PVNH5 $\left\{V_{N2}\right\}$: RASA1${}_{89}$ CCNH${}_{74}$ **MEF2C**${}_{70}$ POLR3G${}_{17}$ COX7C${}_{15}$ MIR9-2${}_{7}$ EDIL3${}_{3}$		
5q33.1	(**RPS14**) ${}^{†‡}$	Dysarthria [22]
**OMIM:** 153550 **Names:** Chromosome 5q deletion syndrome; 5q syndrome; refractory macrocytic anemia due to 5q deletion; MAR $\left\{V_{N2}\right\}$: **RPS14**${}_{29}$ GPX3${}_{18}$ DCTN4${}_{13}$ SPARC${}_{12}$ SLC36A1${}_{12}$ NMUR2${}_{11}$ CD74${}_{10}$ NDST1${}_{9}$ GM2A${}_{8}$ TNIP1${}_{6}$ (7more…) SYNPO${}_{1}$ IRGM${}_{1}$		
5q35.3	(**NSD1**) ${}^{‡}$	Normal [23] or delayed speech
**OMIM:** 117550 **Names:** Sotos syndrome 1; Sotos1; Nevo syndrome; cerebral gigantism, Nevo type; chromosome 5q35 deletion syndrome $\left\{V_{N1}\right\}$ **NSD1**${}_{8}$ $\left\{V_{N2}\right\}$: MAPK9${}_{167}$ LTC4S${}_{53}$ F12${}_{33}$ SQSTM1${}_{30}$ MAML1${}_{29}$ RACK1${}_{25}$ FLT4${}_{23}$ CANX${}_{22}$ GRK6${}_{20}$ GRM6${}_{16}$ (37more…)CLK4${}_{1}$		
**Chromosome 6 deletions**		
6pter–p24	(Multiple incl. **FOXC1**, **GMDS**) ${}^{†‡}$	Delayed speech
**OMIM:** 612582; Cytogenetic location: 6pter–p24 Genomic coordinates (GRCh38): 6:0–13,400,000 **Names:** Chromosome 6pter-p24 deletion syndrome $\left\{V_{N2}\right\}$: RIPK1${}_{83}$ EDN1${}_{43}$ F13A1${}_{34}$ TFAP2A${}_{19}$ DSP${}_{18}$ BMP6${}_{17}$ IRF4${}_{15}$ TUBB2A${}_{14}$ **GMDS**${}_{13}$ ELOVL2${}_{13}$ (31more…) PAK1IP1${}_{1}$ MAK${}_{1}$ FOXQ1${}_{1}$ DUSP22${}_{1}$ C6orf201${}_{1}$		
6q25.3	(Multiple incl. **ARID1B**) ${}^{‡}$	Delayed speech, apraxia, dysarthria [24]
**OMIM:** N.A. $\left\{V_{N2}\right\}$: SLC22A2${}_{53}$ GTF2H5${}_{50}$ ACAT2${}_{49}$ SLC22A1${}_{24}$ EZR${}_{24}$ SOD2${}_{20}$ IGF2R${}_{20}$ SYNJ2${}_{18}$ SLC22A3${}_{16}$ TCP1${}_{13}$ (11more…)		
**Chromosome 7 deletions**		
7p21	(**TWIST1** (7p21.1)) ${}^{‡}$	Delayed speech [25]
**OMIM:** 101400 **Names:** Saethre–Chotzen syndrome; acrocephalosyndactyly III; ACS3; ACS III; Chotzen syndrome; acrocephaly, skull asymmetry, and mild syndactyly $\left\{V_{N2}\right\}$: RPA3${}_{74}$ PRPS1L1${}_{30}$ HDAC9${}_{26}$ ITGB8${}_{18}$ AHR${}_{18}$ POLR1F${}_{16}$ NDUFA4${}_{15}$ DGKB${}_{14}$ **TWIST1**${}_{9}$ THSD7A${}_{4}$ (8more…) MIOS${}_{1}$ MEOX2${}_{1}$ ARL4A${}_{1}$		
7q11.23	(Multiple incl. **ELN**, **LIMK1**, **GTF2IRD1**, **GTF2I**) ${}^{†‡}$	Normal or delayed speech
**OMIM:** 194050; Cytogenetic location: 7q11.23 Genomic coordinates (GRCh38): 7:72,700,000–77,900,000 **Names:** Williams syndrome; WS; WMS; chromosome 7q11.23 deletion syndrome, 1.5- to 1.8-MB; Williams–Beuren syndrome; WBS $\left\{V_{N2}\right\}$: YWHAG${}_{64}$ POM121${}_{59}$ RFC2${}_{57}$ MDH2${}_{42}$ **LIMK1**${}_{34}$ HSPB1${}_{31}$ STX1A${}_{30}$ FZD9${}_{30}$ NCF1${}_{29}$ POR${}_{15}$ (25more…) ABHD11${}_{1}$		
**Chromosome 8 deletions**		
8p23.1	(**GATA4**)	No significant speech anomaly reports
**OMIM:** 179613 (not exclusive to syndrome) **Names:** Recombinant chromosome 8 syndrome; REC8 syndrome; chromosome 8q22.1-qter duplication and 8pter-p23.1 deletion; San Luis Valley syndrome $\left\{V_{N2}\right\}$: FDFT1${}_{44}$ CTSB${}_{17}$ BLK${}_{17}$ TNKS${}_{16}$ **GATA4**${}_{15}$ AGPAT5${}_{14}$ ANGPT2${}_{12}$ MIR124-1${}_{10}$ NEIL2${}_{9}$ CLDN23${}_{8}$ (39more…) PINX1${}_{1}$ MIR598${}_{1}$		
8q22.1	(**CCNE2**, TMEM67, FAM92A1)	Delayed speech
**OMIM:** 608156; Cytogenetic location: 8q22.1 Genomic coordinates (GRCh38): 8:92,300,000–97,900,000 **Names:** Nablus mask-like facial syndrome; NMLFS; chromosome 8q22.1 deletion syndrome $\left\{V_{N2}\right\}$: **CCNE2**${}_{45}$ SDC2${}_{34}$ TP53INP1${}_{13}$ GDF6${}_{12}$ UQCRB${}_{11}$ PTDSS1${}_{9}$ ESRP1${}_{8}$ PDP1${}_{5}$ NDUFAF6${}_{5}$ CDH17${}_{4}$ (3more…)		
8q24.11-q24.13	(TRPS1, **EXT1**) ${}^{‡}$	Delayed speech
**OMIM:** 150230; Cytogenetic location: 8q24.11–q24.13 Genomic coordinates (GRCh38): 8:116,700,000–126,300,000 **Names:** Langer–Giedion syndrome; LGS; chromosome 8q24.1 deletion syndrome; trichorhinophalangeal syndrome type II; TRPS2 $\left\{V_{N2}\right\}$: SQLE${}_{43}$ TAF2${}_{20}$ RAD21${}_{20}$ MIR3610${}_{19}$ TNFRSF11B${}_{15}$ DERL1${}_{13}$ NDUFB9${}_{12}$ **EXT1**${}_{12}$ SLC30A8${}_{10}$ FBXO32${}_{8}$ (11more…) WASHC5${}_{1}$ FAM91A1${}_{1}$ DSCC1${}_{1}$		
**Chromosome 9 deletions**		
9p24.3	(DMRT1, DMRT2) ${}^{†‡}$	Delayed speech, dysarthria, apraxia [26]
**OMIM:** 154230; Cytogenetic location: 9p24.3 Genomic coordinates (GRCh38): 9:0–2,200,000 **Names:** 46,XY sex reversal 4; SRXY4; 46,XY gonadal dysgenesis, partial or complete, with 9p24.3 deletion; chromosome 9p24.3 deletion syndrome $\left\{V_{N1}\right\}$ SMARCA2${}_{9}$ $\left\{V_{N2}\right\}$: SMARCA2${}_{9}$ DOCK8${}_{3}$ WASHC1${}_{1}$		
9q34.3	(**EHMT1**) ${}^{‡}$	Delayed speech [27], Apraxia [28] and Absent speech
OMIM: 610253 Names: Kleefstra syndrome; 9q syndrome; 9q subtelometric deletion syndrome; chromosome 9q34.3 deletion syndrome $\left\{V_{N1}\right\}$ NOTCH1${}_{62}$ $\left\{V_{N2}\right\}$: GRIN1${}_{139}$ TRAF2${}_{78}$ NOTCH1${}_{62}$ PTGDS${}_{51}$ ANAPC2${}_{37}$ TUBB4B${}_{28}$ NELFB${}_{27}$ ENTPD8${}_{25}$ CACNA1B${}_{20}$ AGPAT2${}_{20}$ (36more…) UAP1L1${}_{1}$ MIR602${}_{1}$ FUT7${}_{1}$ CYSRT1${}_{1}$		
**Chromosome 10 deletions**		
10pter–p13 or 10p14–p15.1	(Multiple incl. **GATA3**)	Sensorineural hearing loss
**OMIM:** 146255 **Names:** Barakat syndrome; hypoparathyroidism, sensorineural deafness, and renal disease/dysplasia syndrome; HDRS; nephrosis, nerve deafness, and hypoparathyroidism $\left\{V_{N2}\right\}$: IL2RA${}_{83}$ PRKCQ${}_{71}$ AKR1C3${}_{70}$ IDI1${}_{40}$ CALML5${}_{36}$ AKR1C4${}_{35}$ CALML3${}_{33}$ **GATA3**${}_{27}$ NUDT5${}_{22}$ TAF3${}_{21}$ (39more…) ZMYND11${}_{1}$ CELF2${}_{1}$		
10q23	(**PTEN**, **BMPR1A**)	Delayed or absent speech; impaired speech [29]
OMIM: 612242 (10q22.3–q23.2) Names: Chromosome 10q22.3–q23.2 deletion syndrome $\left\{V_{N1}\right\}$ **PTEN**${}_{107}$ $\left\{V_{N2}\right\}$: **PTEN**${}_{107}$ CYP2C8${}_{100}$ NRG3${}_{85}$ CYP2C9${}_{78}$ FAS${}_{70}$ CYP2C19${}_{66}$ GLUD1${}_{46}$ LIPA${}_{39}$ **BMPR1A**${}_{28}$ PLCE1${}_{25}$ (49more…) PDLIM1${}_{1}$ PCGF5${}_{1}$ MMRN2${}_{1}$ IFIT2${}_{1}$		
10q26	(HMX3, **DOCK1**, C10ORF90) ${}^{†‡}$	Impaired speech; delayed speech [30]
**OMIM:** 609625; Cytogenetic location: 10q26 Genomic coordinates (GRCh38): 10:128,800,000–133,797,422 **Names:** Chromosome 10q26 deletion syndrome; terminal chromosome 10q26 deletion syndrome $\left\{V_{N2}\right\}$: FGFR2${}_{113}$ CYP2E1${}_{91}$ ECHS1${}_{88}$ ACADSB${}_{41}$ **DOCK1**${}_{33}$ BUB3${}_{28}$ OAT${}_{21}$ RPL21P16${}_{20}$ EIF3A${}_{19}$ GRK5${}_{17}$ (38more…) RAB11FIP2${}_{1}$ MKI67${}_{1}$ EBF3${}_{1}$ CUZD1${}_{1}$ CALY${}_{1}$		
**Chromosome 11 deletions**		
11p11.2–p12	(**EXT2**, ALX4) ${}^{‡}$	Idiosyncratic speech, dysarthria, delayed speech, apraxia [31]
**OMIM:** 601224 (11p11.2); Cytogenetic location: 11p11.2 Genomic coordinates (GRCh38): 11:43,400,000–48,800,000 **Names:** Potocki–Shaffer syndrome; PSS; chromosome 11p11.2 deletion syndrome: proximal 11p deletion syndrome; DEFECT11 syndrome $\left\{V_{N2}\right\}$: TRAF6${}_{134}$ NUP160${}_{88}$ F2${}_{85}$ PSMC3${}_{30}$ NR1H3${}_{26}$ CREB3L1${}_{26}$ CKAP5${}_{25}$ DDB2${}_{22}$ SPI1${}_{19}$ HSD17B12${}_{19}$ (36more…)		
11p13-p12	(Multiple incl. WT1, **PAX6**, BDNF) ${}^{†‡}$ (**SLC1A2**, **PRRG4**)${}^{★}$,	Impaired speech [32]
**OMIM:** 612469; Cytogenetic location: 11p13–p12 Genomic coordinates (GRCh38): 11:31,000,000–43,400,000 **Names:** Wilms tumor, aniridia, genitourinary anomalies, mental retardation and obesity syndrome; WAGRO; WAGRO syndrome; WAGR syndrome with obesity; chromosome 11p13-p12 deletion syndrome $\left\{V_{N2}\right\}$: TRAF6${}_{134}$ CAT${}_{37}$ CD44${}_{27}$ CD59${}_{19}$ CSTF3${}_{16}$ **SLC1A2**${}_{14}$ EIF3M${}_{14}$ PDHX${}_{12}$ WT1${}_{10}$ APIP${}_{10}$ (13more…) PRR5L${}_{1}$ ELF5${}_{1}$		
11p15.5	(Multiple incl. CDKN1C, H19, **IGF2**) ${}^{†‡}$	Impaired speech [33]
**OMIM:** 130650; **Names:** 130650: Beckwith–Wiedemann syndrome; BWS; exomphalos–macroglossia–gigantism syndrome; EMG syndrome; Wiedemann–Beckwith syndrome; WBS $\left\{V_{N1}\right\}$: HRAS${}_{347}$ $\left\{V_{N2}\right\}$: HRAS${}_{347}$ INS${}_{103}$ POLR2L${}_{85}$ KCNQ1${}_{69}$ DUSP8${}_{53}$ TNNT3${}_{52}$ TNNI2${}_{52}$ AP2A2${}_{47}$ IRF7${}_{39}$ TH${}_{30}$ (40more…) RIC8A${}_{1}$ RASSF7${}_{1}$ NLRP6${}_{1}$ MIR483${}_{1}$ KRTAP5-4${}_{1}$ KRTAP5-1${}_{1}$ DEAF1${}_{1}$ CRACR2B${}_{1}$ BRSK2${}_{1}$		
11q13.3	(**FGF4**, **FGF3**, **FADD**) ${}^{†‡}$	Delayed speech [34]; Impaired speech [35]
**OMIM:** 166750 **Names:** Chromosome 11q13 deletion syndrome; ododental syndrome; otodental dysplasia and coloboma due to 11q13.3 microdeletion $\left\{V_{N2}\right\}$: **FGF4**${}_{135}$ CCND1${}_{122}$ **FGF3**${}_{103}$ FGF19${}_{95}$ **FADD**${}_{73}$ CPT1A${}_{38}$ CTTN${}_{22}$ PPFIA1${}_{8}$ TPCN2${}_{5}$ SHANK2${}_{4}$ ANO1${}_{3}$		
11q23	(Multiple incl. FLI1) ${}^{†‡}$	Dysarthric speech, absent speech [36]; impaired speech [37]
**OMIM:** 188025; Cytogenetic location: 11q23 Genomic coordinates (GRCh38): 11:114,600,000–121,300,000 **Names:** Chromosome 11q23 deletion syndrome; thrombocytopenia, Paris–Trousseau type; TCPT; Paris–Trousseau syndrome; 11q terminal deletion syndrome $\left\{V_{N2}\right\}$: FXYD2${}_{158}$ PPP2R1B${}_{145}$ CBL${}_{104}$ H2AX${}_{62}$ NCAM1${}_{56}$ CD3G${}_{43}$ CD3D${}_{40}$ SC5D${}_{39}$ DLAT${}_{39}$ APOA1${}_{39}$ (67more…) ZPR1${}_{1}$ TMPRSS4${}_{1}$ TMPRSS13${}_{1}$ TAGLN${}_{1}$ SIK3${}_{1}$ POU2F3${}_{1}$ MPZL2${}_{1}$ C11orf52${}_{1}$		
11q23.3-q25	(**FLI1**, BSX, NRGN, FRA11B, **JAM3**) ${}^{†‡}$	Impaired speech, delayed speech, apraxia [38]
**OMIM:** 147791; Cytogenetic location: 11q23.3–q25 Genomic coordinates (GRCh38): 11:114,600,000–135,086,622 **Names:** Jacobsen syndrome; JBS; Del(11)(qter); distal deletion of 11q; distal monosomy 11q; monosomy 11qter $\left\{V_{N2}\right\}$: FXYD2${}_{158}$ CBL${}_{104}$ KCNJ5${}_{70}$ H2AX${}_{62}$ HSPA8${}_{51}$ CHEK1${}_{48}$ CD3G${}_{43}$ CD3D${}_{40}$ SC5D${}_{39}$ APOA1${}_{39}$ (109more…) ZPR1${}_{1}$ VPS26B${}_{1}$ TMPRSS4${}_{1}$ TMPRSS13${}_{1}$ TAGLN${}_{1}$ SLC37A2${}_{1}$ SIK3${}_{1}$ POU2F3${}_{1}$ MPZL2${}_{1}$ IGSF9B${}_{1}$ GRAMD1B${}_{1}$ EI24${}_{1}$		
**Chromosome 12 deletions**		
12q14.3	(**HMGA2**) ${}^{†‡}$	Absent, impaired [38] or delayed speech [39]
**OMIM:** 618908 **Names:** Silver–Russell syndrome 5; SRS5 $\left\{V_{N2}\right\}$: IRAK3${}_{18}$ WIF1${}_{15}$ MIR6502${}_{15}$ GNS${}_{15}$ MSRB3${}_{12}$ LEMD3${}_{10}$ GRIP1${}_{9}$ **HMGA2**${}_{7}$ CAND1${}_{7}$		
**Chromosome 13 deletions**		
13q12.3	(POMP) ${}^{†‡}$	Delayed speech [40]
**OMIM:** 601952 **Names:** Keratosis linearis with ichthyosis congenita and sclerosing keratoderma syndrome; KLICK syndrome $\left\{V_{N2}\right\}$: HMGB1${}_{43}$ FLT1${}_{36}$ ALOX5AP${}_{13}$ SLC7A1${}_{11}$ HSPH1${}_{11}$ B3GLCT${}_{5}$		
13q14	(**RB1**) ${}^{‡}$	Normal speech [41]
OMIM: 613884; Cytogenetic location: 13q14 Genomic coordinates (GRCh38): 13:50,300,000–54,700,000 Names: Chromosome 13q14 deletion syndrome $\left\{V_{N2}\right\}$: **RB1**${}_{94}$ FOXO1${}_{91}$ GTF2F2${}_{51}$ KBTBD7${}_{50}$ SLC25A15${}_{24}$ HTR2A${}_{23}$ TNFSF11${}_{18}$ LPAR6${}_{16}$ CYSLTR2${}_{15}$ DGKH${}_{14}$ (36more…) WDFY2${}_{1}$ ITM2B${}_{1}$ DLEU2${}_{1}$ DLEU1${}_{1}$ AKAP11${}_{1}$		
13q22.3	(**EDNRB**) ${}^{†‡}$	Normal speech [41]
**OMIM:** 277580; **Names:** Waardenburg syndrome, Type 4A; WS4A; Waardenburg syndrome with Hirschsprung disease, Type 4A; Waardenburg–Shah syndrome; Shah–Waardenburg syndrome; WS4 $\left\{V_{N2}\right\}$: **EDNRB**${}_{26}$ FBXL3${}_{10}$ MYCBP2${}_{1}$		
13q33–q34	(**SOX1**, **ARHGEF7**) ${}^{†‡}$	Apraxia
OMIM: 619148; Cytogenetic location: 13q33–q34 Genomic coordinates (GRCh38): 13:106,400,000–114,364,328 Names: Chromosome 13q33–q34 deletion syndrome $\left\{V_{N2}\right\}$: IRS2${}_{108}$ RASA3${}_{52}$ TFDP1${}_{48}$ F7${}_{46}$ F10${}_{45}$ CDC16${}_{37}$ COL4A1${}_{36}$ COL4A2${}_{33}$ **ARHGEF7**${}_{31}$ ATP4B${}_{27}$ (20more…) ING1${}_{1}$		
**Chromosome 14 deletions**		
14q11–q22	(Multiple incl. **PAX9**, **SUPT16H**, **CHD8**, **RALGAPA1**] ${}^{†‡}$	Delayed [42] or Absent speech
OMIM: 613457; Cytogenetic location: 14q11–q22 Genomic coordinates (GRCh38): 14:18,200,000–57,600,000 **Names:** Chromosome 14q11-q22 deletion syndrome $\left\{V_{N2}\right\}$: NFKBIA${}_{168}$ ADCY4${}_{99}$ GNG2${}_{89}$ SOS2${}_{75}$ POLE2${}_{47}$ PNP${}_{45}$ SLC7A7${}_{40}$ SLC7A8${}_{39}$ BMP4${}_{33}$ GZMB${}_{28}$ (163more…) ZNF219${}_{1}$ WDHD1${}_{1}$ TRD${}_{1}$ TEP1${}_{1}$ **RALGAPA1**${}_{1}$ **PAX9**${}_{1}$ NDRG2${}_{1}$ MIR208A${}_{1}$ DLGAP5${}_{1}$ CEBPE${}_{1}$ BAZ1A${}_{1}$ AKAP6${}_{1}$		
14q22.1–q23.1	(Multiple incl. **PTGDR**, **BMP4**) ${}^{‡}$	Impaired speech [43]
OMIM: 609640; Cytogenetic location: 14q22.1–q22.3 Genomic coordinates (GRCh38): 14:50,400,000–57,600,000 Names: Frias syndrome; Chromosome 14q22 deletion syndrome; growth deficiency, facial anomalies, and brachydactyly $\left\{V_{N2}\right\}$: GNG2${}_{89}$ MNAT1${}_{70}$ PRKCH${}_{35}$ **BMP4**${}_{33}$ PSMA3${}_{26}$ GNPNAT1${}_{25}$ PELI2${}_{24}$ PPM1A${}_{23}$ PYGL${}_{21}$ PTGER2${}_{20}$ (31more…) WDHD1${}_{1}$ SIX6${}_{1}$ SIX1${}_{1}$ DLGAP5${}_{1}$		
14q32.2	(**DLK1**, **MEG3**, RTL1) ${}^{‡}$	Delayed speech; idiosyncratic speech [44]
OMIM: 608149 (paternal)/ 616222 (maternal); Cytogenetic location: 14q32 Genomic coordinates (GRCh38): 14:103,500,000–107,043,718 Names: 14q32.2 Kagami–Ogata syndrome; uniparental disomy, paternal, chromosome 14/14q32.2; Temple syndrome; uniparental disomy, maternal, chromosome 14 $\left\{V_{N2}\right\}$: BDKRB2${}_{32}$ CCNK${}_{25}$ BDKRB1${}_{24}$ YY1${}_{21}$ MIR6764${}_{20}$ CYP46A1${}_{19}$ PAPOLA${}_{18}$ DEGS2${}_{13}$ EVL${}_{12}$ WARS1${}_{10}$ (12more…) MIR345${}_{1}$ MIR342${}_{1}$ **MEG3**${}_{1}$		
**Chromosome 15 deletions**		
15q11.2	(**NIPA1**, **NIPA2**, **CYFIP1**, **TUBGCP5**) ${}^{†‡}$	Delayed speech
**OMIM:** 615656; Cytogenetic location: 15q11.2 Genomic coordinates (GRCh38): 15:20,500,000–25,500,000 **Names:** Burnside–Butler syndrome; 15q11.2 BP1–BP2 microdeletion $\left\{V_{N2}\right\}$: **CYFIP1**${}_{16}$ UBE3A${}_{15}$ **TUBGCP5**${}_{7}$ SNURF${}_{7}$ OR4N4${}_{6}$ SNRPN${}_{5}$ OR4N4C${}_{5}$ OR4M2${}_{5}$ **NIPA2**${}_{2}$ **NIPA1**${}_{2}$ NDN${}_{2}$		
15q11–q13	(**NDN**, **SNRPN**) ${}^{†‡}$	Delayed or Impaired speech
**OMIM:** 176270 (for 5q11.2) **Names:** Prader-Willi syndrome; Prader-Lambhart-Willi syndrome; Labhart-Willi syndrome; Prader’s syndrome; Prader-Labhart-Willi-Fanconi syndrome $\left\{V_{N2}\right\}$: HERC2${}_{22}$ TJP1${}_{20}$ RYR3${}_{16}$ GABRA5${}_{16}$ CYFIP1${}_{16}$ **UBE3A**${}_{15}$ GABRB3${}_{15}$ CHRNA7${}_{14}$ GABRG3${}_{13}$ TUBGCP5${}_{7}$ (20more…) MTMR10${}_{1}$ HMGN2P5${}_{1}$ FMN1${}_{1}$		
15q11–q13	(**UBE3A**) ${}^{†‡}$	Absent speech; impaired speech [45]
**OMIM:** 105830 (for 15q11.2) **Names:** Angelman syndrome; happy puppet syndrome –Same as above–		
15q13.3	(**CHRNA7**, CHRFAM7A, **OTUD7A**) ${}^{†‡}$	Impaired or idiosyncratic speech
**OMIM:** 612001; Cytogenetic location: 15q13.3 Genomic coordinates (GRCh38): 15:30,900,000–33,400,000 **Names:** Chromosome 15q13.3 microdeletion syndrome $\left\{V_{N2}\right\}$: RYR3${}_{16}$ **CHRNA7**${}_{14}$ TRPM1${}_{4}$ OTUD7A${}_{4}$ FAN1${}_{4}$ ARHGAP11A${}_{3}$ MTMR10${}_{1}$ FMN1${}_{1}$		
15q24	(Multiple incl. **SIN3A**) ${}^{†‡}$	Delayed or impaired speech
**OMIM:** 613406 **Names:** Witteveen–Kolk syndrome; WITKOS; chromosome 15q24 deletion syndrome (included) $\left\{V_{N2}\right\}$: CSK${}_{89}$ NRG4${}_{82}$ CYP1A2${}_{79}$ HCN4${}_{56}$ CYP1A1${}_{48}$ PML${}_{32}$ CYP11A1${}_{32}$ **SIN3A**${}_{25}$ COX5A${}_{16}$ MPI${}_{15}$ (28more…) SNX33${}_{1}$ PTPN9${}_{1}$ CLK3${}_{1}$		
**Chromosome 16 deletions**		
16p11.2	(**SH2B1** ${}^{‡}$, **TBX6**, **CORO1A**) ${}^{†‡}$	Apraxia [46]; dysarthria [47]; delayed or impaired speech
OMIM: 611913; Cytogenetic location: 16p11.2 Genomic coordinates (GRCh38): 16:28,500,000–35,300,000 Names: Chromosome 16p11.2 deletion syndrome (593 kb) or 16p11.2 deletion syndrome (220 kb) $\left\{V_{N1}\right\}$ SRCAP${}_{1}$ $\left\{V_{N2}\right\}$: MAPK3${}_{447}$ ALDOA${}_{46}$ CD19${}_{38}$ ITGAM${}_{32}$ VKORC1${}_{31}$ SULT1A1${}_{29}$ ITGAL${}_{25}$ STX4${}_{22}$ PYCARD${}_{19}$ CDIPT${}_{19}$ (60more…) SRCAP${}_{1}$ RNF40${}_{1}$ PYDC1${}_{1}$ ORAI3${}_{1}$ MAZ${}_{1}$ HIRIP3${}_{1}$ AHSP${}_{1}$		
16p12.2–p11.2	(Multiple incl. **SH2B1**) ${}^{*}$ [76]	Delayed or impaired speech
**OMIM:** 613604; Cytogenetic location: 16p12.2–p11.2 Genomic coordinates (GRCh38): 16:21,200,000–35,300,000 **Names:** Chromosome 16p12.2–p11.2 deletion syndrome, 7.1 to 8.7 MB $\left\{V_{N1}\right\}$ SRCAP${}_{1}$ $\left\{V_{N2}\right\}$: MAPK3${}_{447}$ PRKCB${}_{184}$ PLK1${}_{65}$ ALDOA${}_{46}$ CD19${}_{38}$ SCNN1G${}_{35}$ SCNN1B${}_{35}$ TNRC6A${}_{33}$ ITGAM${}_{32}$ VKORC1${}_{31}$ (96more…) XPO6${}_{1}$ USP31${}_{1}$ SRCAP${}_{1}$ RNF40${}_{1}$ PYDC1${}_{1}$ ORAI3${}_{1}$ MAZ${}_{1}$ IGSF6${}_{1}$ HIRIP3${}_{1}$ CLN3${}_{1}$ CHP2${}_{1}$ AHSP${}_{1}$		
16p12.1	(Multiple) ${}^{‡}$	Delayed speech
**OMIM:** 136570; Cytogenetic location: 16p12 Genomic coordinates (GRCh38): 16:24,200,000–28,500,000 **Names:** Chromosome 16p12.1 deletion syndrome, 520 KB $\left\{V_{N2}\right\}$: TNRC6A${}_{33}$ IL4R${}_{24}$ CACNG3${}_{20}$ EIF3CL${}_{14}$ HS3ST4${}_{8}$ GTF3C1${}_{8}$ SLC5A11${}_{7}$ IL27${}_{7}$ AQP8${}_{7}$ NSMCE1${}_{5}$ (7more…) XPO6${}_{1}$ CLN3${}_{1}$		
16p13.11	(Multiple incl. **MYH11**) ${}^{†}$	Delayed speech [48]
**OMIM:** 619351 **Names:** 16p13.1 microdeletion predisposing to autism and/or ID; megacystis–microcolon–intestinal hypoperistalsis syndrome 2; MMIHS2 $\left\{V_{N2}\right\}$: ABCC1${}_{41}$ NDE1${}_{23}$ MIR484${}_{23}$ **MYH11**${}_{18}$ RRN3${}_{7}$ ABCC6${}_{4}$		
16p13.3	(**CREBBP**, DNASE1, **TRAP1**) ${}^{†‡}$	Apraxia, dysarthria, impaired or delayed speech [49]
**OMIM:** 610543; Cytogenetic location: 16p13.3 Genomic coordinates (GRCh38): 16:0–7,800,000 **Names:** Chromosome 16p13.3 deletion syndrome, proximal; severe Rubinstein–Taybi syndrome (RTS); broad thumb–hallux syndrome; Rubinstein syndrome; Rubinstein–Taybi deletion syndrome; RSTS deletion syndrome $\left\{V_{N1}\right\}$ **CREBBP**${}_{140}$ $\left\{V_{N2}\right\}$: PDPK1${}_{173}$ **CREBBP**${}_{140}$ ADCY9${}_{102}$ TSC2${}_{78}$ MLST8${}_{69}$ GNG13${}_{68}$ CACNA1H${}_{60}$ AXIN1${}_{49}$ UBE2I${}_{44}$ ELOB${}_{40}$ RPS2${}_{35}$ (111more…) WDR24${}_{1}$ TFAP4${}_{1}$ SOX8${}_{1}$ SEPTIN12${}_{1}$ RHBDL1${}_{1}$ RBFOX1${}_{1}$ PGP${}_{1}$ NPRL3${}_{1}$ NAGPA${}_{1}$ MIR3176${}_{1}$ HAGHL${}_{1}$ GNPTG${}_{1}$ GLIS2${}_{1}$ FAHD1${}_{1}$ E4F1${}_{1}$ CHTF18${}_{1}$ BAIAP3${}_{1}$		
16q22	(**CBFB**) ${}^{†‡}$	–Not available–
**OMIM:** 614541; Cytogenetic location: 16q22 Genomic coordinates (GRCh38): 16:72,800,000–74,100,000 **Names:** Chromosome 16q22 deletion syndrome $\left\{V_{N1}\right\}$: CMTR2${}_{2}$ $\left\{V_{N2}\right\}$: CDH1${}_{60}$ TRADD${}_{50}$ SNTB2${}_{50}$ SLC7A6${}_{42}$ NFATC3${}_{30}$ TAT${}_{29}$ PARD6A${}_{27}$ E2F4${}_{26}$ NQO1${}_{25}$ ST3GAL2${}_{24}$ (83more…) WWP2${}_{1}$ PSKH1${}_{1}$ PKD1L3${}_{1}$ NUTF2${}_{1}$ NOB1${}_{1}$ CHTF8${}_{1}$ CDH16${}_{1}$		
16q24.3–q24.2	(**CDH15**, **ZNF778**, ANKRD11, **ZFPM1**) ${}^{**}$	Delayed or impaired speech [50]
$\left\{V_{N2}\right\}$: SLC7A5${}_{48}$ MVD${}_{42}$ CYBA${}_{28}$ APRT${}_{28}$ RPL13${}_{22}$ CDT1${}_{20}$ TUBB3${}_{13}$ MC1R${}_{11}$ DPEP1${}_{10}$ TRAPPC2L${}_{9}$ (18more…) SPIRE2${}_{1}$ CHMP1A${}_{1}$		
**Chromosome 17 deletions**		
17p11.2	(**LLGL1**, RAI1, **UBB**) ${}^{†‡}$	Delayed speech; dysarthria [51]
**OMIM:** 182290 **Names:** Smith–Magenis syndrome; chromosome 17p11.2 deletion syndrome $\left\{V_{N2}\right\}$: **UBB**${}_{280}$ MAP2K3${}_{108}$ MAPK7${}_{72}$ ALDH3A1${}_{50}$ SHMT1${}_{49}$ ALDH3A2${}_{46}$ SREBF1${}_{27}$ TOP3A${}_{25}$ MIR6778${}_{20}$ KCNJ12${}_{17}$ (26more…)		
17p13.1	(Multiple incl. **KCNAB3**, **GUCY2D**, **TP53**, **TRAPPC1**, **MPDU1**, CDG1F, **FXR2**, FMRP, **EFNB3**) ${}^{†‡}$	Absent speech
**OMIM:** 613776; Cytogenetic location: 17p13.1 Genomic coordinates (GRCh38): 17:6,500,000–10,800,000 Names: Chromosome 17p13.1 deletion syndrome $\left\{V_{N1}\right\}$ **TP53**${}_{206}$ KDM6B${}_{8}$ $\left\{V_{N2}\right\}$: **TP53**${}_{206}$ ATP1B2${}_{161}$ PIK3R5${}_{104}$ DLG4${}_{76}$ POLR2A${}_{71}$ ALOX12${}_{55}$ ALOX15B${}_{51}$ ALOX12B${}_{48}$ DVL2${}_{45}$ SLC2A4${}_{38}$ VAMP2${}_{37}$ (63more…) TNK1${}_{1}$ SHBG${}_{1}$ MYH4${}_{1}$ MYH13${}_{1}$ MYH1${}_{1}$ MIR497${}_{1}$ MIR324${}_{1}$ DNAH2${}_{1}$		
17p13.3	(LIS1, **PAFAH1B1**, **YWHAE**) †‡	Delayed speech [52]
**OMIM:** 247200; Cytogenetic location: 17p13.3 Genomic coordinates (GRCh38): 17:0–3,400,000 **Names:** Miller–Dieker lissencephaly syndrome; MDLS; chromosome 17p13.3 deletion syndrome (included) $\left\{V_{N2}\right\}$: CRK${}_{98}$ RPA1${}_{86}$ **YWHAE**${}_{83}$ **PAFAH1B1**${}_{34}$ INPP5K${}_{16}$ ABR${}_{12}$ SERPINF2${}_{10}$ SLC43A2${}_{9}$ OR3A1${}_{8}$ OR1G1${}_{8}$ (27more…) SMYD4${}_{1}$ SERPINF1${}_{1}$		
17q11.2	(**NF1**)‡	No significant speech issues
**OMIM:** 162200 **Names:** Neurofibromatosis type I; NF1; Von Recklinghausen disease; neurofibromatosis, peripheral type; Morbus–Recklinghausen $\left\{V_{N2}\right\}$: SLC6A4${}_{73}$ KSR1${}_{66}$ **NF1**${}_{62}$ NOS2${}_{41}$ PSMD11${}_{30}$ VTN${}_{28}$ NLK${}_{27}$ CDK5R1${}_{26}$ RPL23A${}_{22}$ ALDOC${}_{19}$ (30more…) RAB11FIP4${}_{1}$ MIR451A${}_{1}$ MIR423${}_{1}$		
17q12	(Multiple incl. **HNF1B**, **LHX1**, **CCL3L3**, SNIP) ${}^{†‡}$	Delayed or impaired speech
**OMIM:** 614527; Cytogenetic location: 17q12 Genomic coordinates (GRCh38): 17:33,500,000–39,800,000 **Names:** Chromosome 17q12 deletion syndrome $\left\{V_{N1}\right\}$ ERBB2${}_{124}$ $\left\{V_{N2}\right\}$: CACNB1${}_{129}$ ERBB2${}_{124}$ AP2B1${}_{49}$ PIP4K2B${}_{43}$ ACACA${}_{41}$ CCL5${}_{30}$ CCL2${}_{30}$ PSMB3${}_{25}$ CCL4${}_{24}$ RPL23${}_{23}$ (49more…) PEX12${}_{1}$ MMP28${}_{1}$ DUSP14${}_{1}$		
17q21.31	(**KANSL1**, **MAPT**, **CRHR1**) ${}^{‡}$	Delayed or absent speech
**OMIM:** 610443 **Names:** Koolen–De Vries syndrome; KDVS; chromosome 17q21.31 deletion syndrome; Microdeletion 17q21.31 syndrome $\left\{V_{N1}\right\}$: **KANSL1**${}_{4}$ BRCA1${}_{64}$ $\left\{V_{N2}\right\}$: ITGA2B${}_{95}$ BRCA1${}_{64}$ MAP3K14${}_{59}$ G6PC1${}_{43}$ FZD2${}_{38}$ WNT3${}_{36}$ DUSP3${}_{31}$ HDAC5${}_{28}$ NSF${}_{27}$ AOC3${}_{24}$ (36more…) TMEM106A${}_{1}$ RND2${}_{1}$ HEXIM1${}_{1}$		
17q23.1-q23.2	(TBX4) ${}^{†‡}$	Delayed speech [53]
**OMIM:** 613355; Cytogenetic location: 17q23.1–q23.2 Genomic coordinates (GRCh38): 17:59,500,000–63,100,000 **Names:** Chromosome 17q23.1–q23.2 deletion syndrome $\left\{V_{N2}\right\}$: RPS6KB1${}_{91}$ CLTC${}_{54}$ BRIP1${}_{24}$ MIR21${}_{19}$ SKA2${}_{12}$ MRC2${}_{12}$ PTRH2${}_{7}$ CA4${}_{7}$ MED13${}_{5}$ DHX40${}_{4}$ (5more…) BCAS3${}_{1}$ APPBP2${}_{1}$		
17q24.3–q24.2	(Multiple incl. **ABCA5**, **MAP2K6**, **SOX9**) ${}^{†‡}$	Delayed speech [54]
OMIM: 135400; Cytogenetic location: 17q24.2–q24.3 Genomic coordinates (GRCh38): 17:66,200,000–72,900,000 Names: Hypertrichosis, congenital generalized, with or without gingival hyperplasia; HTC3; fibromatosis, gingival, with hypertrichosis; chromosome 17q24.2–q24.3 deletion syndrome $\left\{V_{N2}\right\}$: PRKCA${}_{237}$ **MAP2K6**${}_{131}$ PRKAR1A${}_{122}$ KCNJ2${}_{71}$ PSMD12${}_{31}$ CACNG4${}_{21}$ KPNA2${}_{16}$ KCNJ16${}_{13}$ (14more…) BPTF${}_{1}$		
**Chromosome 18 deletions**		
18q	(Multiple incl. **MBP**, TSHZ1) ${}^{†‡}$	Delayed [55] or impaired [56] speech
**OMIM:** 601808; Cytogenetic location: 18q Genomic coordinates (GRCh38): 18:18,500,000–80,373,285 **Names:** Chromosome 18q deletion syndrome; 18q syndrome $\left\{V_{N2}\right\}$: BCL2${}_{100}$ SMAD4${}_{79}$ ROCK1${}_{78}$ SMAD2${}_{66}$ ACAA2${}_{60}$ NFATC1${}_{52}$ PIK3C3${}_{43}$ SMAD7${}_{36}$ LAMA3${}_{35}$ SLC14A2${}_{34}$ (112more…) SS18${}_{1}$ SETBP1${}_{1}$ SERPINB4${}_{1}$ SERPINB13${}_{1}$ RIOK3${}_{1}$ NETO1${}_{1}$ MIR122${}_{1}$ MBD1${}_{1}$ LINC-ROR${}_{1}$ GAREM1${}_{1}$ CELF4${}_{1}$		
**Chromosome 19 deletions**		
19p13.13	(Multiple incl. NFIX, MAST1, CALR) ${}^{†‡}$	Delayed speech; impaired speech [57]
**OMIM:** 613638; Cytogenetic location: 19p13.13 Genomic coordinates (GRCh38): 19:12,600,000–13,800,000 **Names:** Chromosome 19p13.13 deletion syndrome $\left\{V_{N1}\right\}$ MAP2K2${}_{257}$ UHRF1${}_{6}$ $\left\{V_{N2}\right\}$: MAP2K2${}_{257}$ FGF22${}_{102}$ VAV1${}_{100}$ POLR2E${}_{85}$ SHC2${}_{81}$ GNG7${}_{75}$ PIP5K1C${}_{58}$ GTF2F1${}_{53}$ GNA11${}_{48}$ PSPN${}_{47}$ (121more…) ZBTB7A${}_{1}$ TJP3${}_{1}$ SEMA6B${}_{1}$ REXO1${}_{1}$ PLIN4${}_{1}$ MYDGF${}_{1}$ MIR7-3${}_{1}$ DPP9${}_{1}$ DAZAP1${}_{1}$		
19q13.11	(Multiple incl. LSM14A, **UBA2**, **WTIP**, TSHZ3) ${}^{†‡}$	Delayed or absent speech; impaired speech [58]
**OMIM:** 613026 (distal)/ 617219 (proximal); Cytogenetic location: 19q13.11 Genomic coordinates (GRCh38): 19:31,900,000–35,100,000 **Names:** Chromosome 19q13.11 deletion syndrome, distal; chromosome 19q13.11 deletion syndrome, proximal $\left\{V_{N1}\right\}$ CEBPA${}_{23}$ $\left\{V_{N2}\right\}$: SCN1B${}_{62}$ GPI${}_{51}$ SLC7A9${}_{39}$ PSMC4${}_{28}$ CEBPA${}_{23}$ SLC7A10${}_{13}$ **UBA2**${}_{8}$ CHST8${}_{7}$ **WTIP**${}_{6}$ RGS9BP${}_{6}$ (12more…)		
**Chromosome 20 deletions**		
20p12.3	(Multiple incl. **BMP2**)	Delayed or Impaired speech [59]
$\left\{V_{N2}\right\}$: PLCB1${}_{159}$ PCNA${}_{79}$ PLCB4${}_{68}$ **BMP2**${}_{33}$ MCM8${}_{25}$ PROKR2${}_{9}$ CRLS1${}_{9}$ CDS2${}_{9}$ GPCPD1${}_{8}$ RPS18P1${}_{6}$ HAO1${}_{6}$ TRMT6${}_{3}$ LRRN4${}_{1}$		
**Chromosome 22 deletions**		
22q11.2	(Multiple incl. **TBX1** ${}^{†}$, **COMT** ${}^{†}$, INI1 ${}^{†‡}$, **TOP3B** ${}^{†‡}$ )	Apraxia, dysarthria, delayed, or impaired speech [60]
**OMIM:** 611867; Cytogenetic location: 22q11.2 Genomic coordinates (GRCh38): 22:23,100,000–25,500,000 **Names:** Chromosome 22q11.2 deletion syndrome; distal chromosome 22q11.2 deletion syndrome $\left\{V_{N2}\right\}$: MAPK1${}_{469}$ CRKL${}_{68}$ GGT1${}_{62}$ BID${}_{56}$ ADORA2A${}_{35}$ **COMT**${}_{33}$ GNAZ${}_{32}$ UPB1${}_{24}$ ATP6V1E1${}_{23}$ IGL${}_{22}$ (56more…) PIWIL3${}_{1}$ MIR185${}_{1}$		
22q12.2	(**NF2**) ${}^{‡}$	Delayed or impaired speech [61]
**OMIM:** 101000 **Names:** Neurofibromatosis type II; neurofibromatosis, central type; acoustic schwannomas, bilateral; bilateral acoustic neurofibromatosis; BANF; acoustic neurinoma, bilateral; ACN $\left\{V_{N2}\right\}$: LIF${}_{26}$ PLA2G3${}_{20}$ AP1B1${}_{20}$ LIMK2${}_{19}$ INPP5J${}_{18}$ OSM${}_{15}$ PISD${}_{14}$ SFI1${}_{13}$ RNF185${}_{13}$ MTMR3${}_{12}$ (17more…) SEC14L2${}_{1}$ PIK3IP1${}_{1}$ MIR3200${}_{1}$ DEPDC5${}_{1}$		
22q13.3	(**ARSA**, **SHANK3**) ${}^{‡}$	Delayed or absent speech
**OMIM:** 606232 **Names:** Phelan–McDermid syndrome; PHMDS; chromosome 22q13.3 deletion syndrome; telomeric 22q13 monosomy syndrome $\left\{V_{N1}\right\}$ BRD1${}_{9}$ $\left\{V_{N2}\right\}$: MAPK11${}_{140}$ MAPK12${}_{84}$ NUP50${}_{79}$ PRR5${}_{38}$ PPARA${}_{35}$ WNT7B${}_{31}$ TYMP${}_{23}$ HDAC10${}_{19}$ CHKB${}_{19}$ **ARSA**${}_{19}$ (35more…) UPK3A${}_{1}$ PLXNB2${}_{1}$ GRAMD4${}_{1}$ CELSR1${}_{1}$		
**Chromosome X deletions**		
Xp11.3	(Multiple incl. **RP2**, ZNF674) ${}^{†‡}$	Impaired speech [62]
**OMIM:** 300578; Cytogenetic location: Xp11.3 Genomic coordinates (GRCh38): X:42,500,000–47,600,000 **Names:** Chromosome Xp11.3 deletion syndrome; mental retardation, X-linked, with retinitis pigmentosa $\left\{V_{N1}\right\}$ KDM6A${}_{6}$ $\left\{V_{N2}\right\}$: ARAF${}_{106}$ MAOA${}_{72}$ MAOB${}_{37}$ UBA1${}_{18}$ TIMP1${}_{16}$ NDUFB11${}_{10}$ MIR221${}_{10}$ CHST7${}_{10}$ MIR222${}_{9}$ USP11${}_{8}$ (10more…)																														
Xp21	(**GK**, **DMD**, **NR0B1**) ^†‡^	Delayed speech [63]
**OMIM:** 300679; Cytogenetic location: Xp21 Genomic coordinates (GRCh38): X:31,500,000–37,800,000 **Names:** Chromosome Xp21 deletion syndrome; Complex glycerol kinase deficiency $\left\{V_{N2}\right\}$: TAB3${}_{52}$ CYBB${}_{26}$ **GK**${}_{16}$ **DMD**${}_{12}$ **NR0B1**${}_{8}$ XK${}_{5}$ ARX${}_{2}$		
Xq28 (a)	(**ABCD1**, **BCAP31**, **SLC6A8**) ${}^{†‡}$	Delayed speech [64]
**OMIM:** 300475 **Names:** Deafness, dystonia, and cerebral hypomyelination; DDCH; contiguous ABCD1/DXS1375E deletion syndrome, included; CADDS, included $\left\{V_{N2}\right\}$: IKBKG${}_{161}$ IRAK1${}_{85}$ MIR718${}_{54}$ DUSP9${}_{52}$ F8${}_{46}$ H2AB1${}_{43}$ NSDHL${}_{35}$ G6PD${}_{33}$ FLNA${}_{28}$ IDH3G${}_{26}$ (54more…) MPP1${}_{1}$ MIR224${}_{1}$ MIR105-1${}_{1}$ MAGEA11${}_{1}$		
Xq28 (b)	(**MECP2**) ${}^{†‡}$	Normal to absent speech
**OMIM:** 312750 **Names:** Rett syndrome; RTT; RTS autism, dementia, ataxia, and loss of purposeful hand use –same as above–			
**Chromosome Y deletions**		
Yq11	(USP9Y, BPY2, CDY1) ${}^{†‡}$	Normal speech
OMIM: 415000 **Names:** Spermatogenic failure, Y-linked, 2; SPGFY2; Sertoli-cell-only syndrome; Del Castillo syndrome; germ cell aplasia, spermatogenic failure $\left\{V_{N2}\right\}$: RPS4Y2${}_{25}$ UTY${}_{4}$ NLGN4Y${}_{4}$ KDM5D${}_{3}$ CD24P4${}_{3}$ TMSB4Y${}_{1}$		

**Sources:** †: [77], ‡: [78], †‡: From OMIM records, ⋆: [32], ∗∗: [50].
